# Metagenomics harvested genus-specific single-stranded DNA-annealing proteins improve and expand recombineering in *Pseudomonas* species

**DOI:** 10.1093/nar/gkad1024

**Published:** 2023-11-06

**Authors:** Enrique Asin-Garcia, Luis Garcia-Morales, Tessa Bartholet, Zhuobin Liang, Farren J Isaacs, Vitor A P Martins dos Santos

**Affiliations:** Laboratory of Systems and Synthetic Biology, Wageningen University & Research, Wageningen 6708 WE, The Netherlands; Bioprocess Engineering Group, Wageningen University & Research, Wageningen 6700 AA, The Netherlands; Laboratory of Systems and Synthetic Biology, Wageningen University & Research, Wageningen 6708 WE, The Netherlands; Laboratory of Systems and Synthetic Biology, Wageningen University & Research, Wageningen 6708 WE, The Netherlands; Department of Molecular, Cellular, and Developmental Biology, Yale University, New Haven, CT 06520, USA; Systems Biology Institute, Yale University, West Haven, CT 06516, USA; Department of Molecular, Cellular, and Developmental Biology, Yale University, New Haven, CT 06520, USA; Systems Biology Institute, Yale University, West Haven, CT 06516, USA; Department of Biomedical Engineering, Yale University, New Haven, CT 06520, USA; Laboratory of Systems and Synthetic Biology, Wageningen University & Research, Wageningen 6708 WE, The Netherlands; Bioprocess Engineering Group, Wageningen University & Research, Wageningen 6700 AA, The Netherlands; LifeGlimmer GmbH, Berlin 12163, Germany

## Abstract

The widespread *Pseudomonas* genus comprises a collection of related species with remarkable abilities to degrade plastics and polluted wastes and to produce a broad set of valuable compounds, ranging from bulk chemicals to pharmaceuticals. *Pseudomonas* possess characteristics of tolerance and stress resistance making them valuable hosts for industrial and environmental biotechnology. However, efficient and high-throughput genetic engineering tools have limited metabolic engineering efforts and applications. To improve their genome editing capabilities, we first employed a computational biology workflow to generate a genus-specific library of potential single-stranded DNA-annealing proteins (SSAPs). Assessment of the library was performed in different *Pseudomonas* using a high-throughput pooled recombinase screen followed by Oxford Nanopore NGS analysis. Among different active variants with variable levels of allelic replacement frequency (ARF), efficient SSAPs were found and characterized for mediating recombineering in the four tested species. New variants yielded higher ARFs than existing ones in *Pseudomonas putida* and *Pseudomonas aeruginosa*, and expanded the field of recombineering in *Pseudomonas taiwanensis*and *Pseudomonas fluorescens*. These findings will enhance the mutagenesis capabilities of these members of the *Pseudomonas* genus, increasing the possibilities for biotransformation and enhancing their potential for synthetic biology applications.

## Graphical Abstract

**Graphical Abstract:**
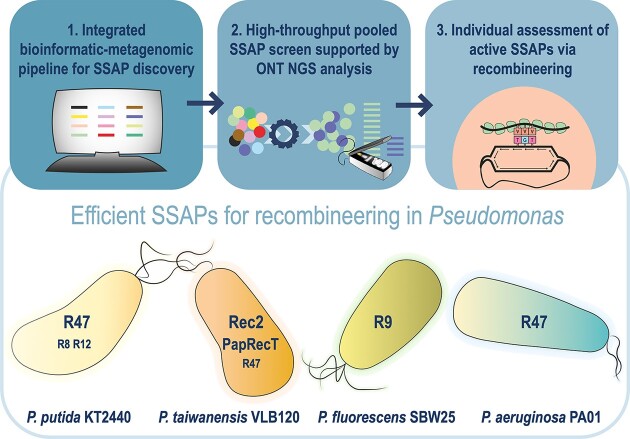

## Introduction

*Pseudomonas* species, with their extensive metabolic diversity and genetic adaptability (1), stand out as exceptionally attractive bacterial chassis for synthetic biology applications (2). Notably, *Pseudomonas putida* KT2440 is known for its proficiency in degrading toxic compounds, high resistance to solvents and antibiotics, and suitability for metabolic engineering (3–8). Notably, *Pseudomonas taiwanensis* VLB120 has gained prominence in recent years due to its excellence as a solvent-tolerant strain equipped with resistance pumps. It exhibits the capacity to grow in aliphatic alcohols and hydrophobic aromatics, and utilizes a wide spectrum of organic molecules as a carbon sources (9–14). The rhizobacterium *Pseudomonas fluorescens* SBW25 is commonly employed as a biocontrol agent, producing secondary metabolites that suppress plant pathogens and shows tolerance to adverse conditions, making it suitable for bioremediation and agricultural applications (15–20). *Pseudomonas aeruginosa* PA01, despite its status as an opportunistic pathogen, is recognised for its rapid acquisition of antibiotic resistances and its production of secondary metabolites with potent antimicrobial properties. These traits make it a valuable asset in medicine, industries and environmental applications (21–25). Collectively, these traits are remarkably valuable for the harsh conditions of industrial bioreactors. These properties motivate the development of genetic engineering tools aimed at studying and harnessing their unique properties.

Although *Pseudomonas* strains have an established role in the biotechnology field (26–28), their potential could be enhanced by further exploiting their unique traits and improving our ability to modify them using genetically engineering (29–31). Although improving intrinsic lifestyle characteristics such as tolerance, handling, growth and substrate consumption rates remains challenging, the incredible progress of genetic engineering during the last decades sets the stage for efficient tailoring of microbial metabolism for a large array of bioconversions, including, for example, the assimilation of non-canonical nutrients and C1 compounds. Currently, the level of development of synthetic biology tools varies among the aforementioned *Pseudomonas* species. The development of a standardized and orthogonal tool set that can be implemented in different *Pseudomonas* would propel their value as promising hosts for the bio-based industry (32,33).

The recent advent of gene editing technologies permits precise manipulation of genomes in a growing number of organisms. Recombination-mediated genetic engineering, or recombineering, is uniquely suited to address key challenges in the field such as genome-scale reverse genetics or the generation of combinatorial genetic diversity at base-pair resolution (34). This efficient bioengineering technique harnesses phage-derived proteins for *in vivo* scarless manipulation of genomes thanks to the integration of single-stranded DNA (ssDNA) (35) or double-stranded DNA (dsDNA) (36) (through an ssDNA intermediate) (37,38) into a replicating chromosome. Single-stranded DNA-annealing proteins (SSAPs), often referred to as recombinases, promote the incorporation of the ssDNA segments during DNA replication making use of their short homology arms. In this way, such DNA segments bearing designed modifications can be inherited and spread across the cell population allowing the generation of genomic deletions, insertions and single-nucleotide substitutions (39).

In *Escherichia coli*, the redβ protein of the λ Red system has long been the standard recombinase for oligonucleotide-mediated recombineering (35). Cycles of recombinase expression during subsequent rounds of DNA segregation can induce multiple random or specific mutations on discrete sites (40,41). Thus, improvement of the recombineering process utilising phage-derived recombinases fostered genome-scale editing in *E. coli* and set the basis for tools like Multiplex Automated Genome Engineering (MAGE) (40), Directed Evolution with Random Genomic mutations (DIvERGE) (42) or retron-recombineering (43,44). The significant impact of these recombineering-based technologies in *E. coli* and other bacteria has been continuously demonstrated in a growing number of projects including single gene evolution (45), metabolism rewiring via pathway diversification (40) or whole-genome recoding (46,47).

Unfortunately, the recombineering power of redβ is not always readily imparted to other bacterial species including the *Pseudomonas*, which implies specificity of the bacteria–phage interaction (48). This apparent host tropism results from the distinct recognition of the bacterial single-stranded DNA-binding protein (SSB) at the carboxy-terminal site by the SSAP (49). Attempts of redβ-analog discovery in *Pseudomonas* species have been pursued for *P. putida* (50,51), *P. aeruginosa* (32,52) and *P. fluorescens* (32) with promising results. Yet, the efficiency of the best candidates has not always been consistent across species or has dropped dramatically when multiplexing resulting in recombineering levels far from those obtained in *E. coli* with redβ (53). While the *P. putida*-borne Rec2 brought about the High-Efficiency Multi-site Genomic Editing system (HEMSE) in its native species (highest reported oligo allelic replacement frequency, ARF = 4.8 × 10^−2^) (53), it quickly became overshadowed by the results of PapRecT in *P. aeruginosa* (highest reported oligo ARF = 1.5 × 10^−1^) (52). Conversely, this recombinase that originates from a *P. aeruginosa* phage and was identified by Serial Enrichment for Efficient Recombineering (SEER) showed improved recombineering frequency in *P. aeruginosa* (52) but exhibited equal or lower activity than Rec2 in *P. putida* (highest reported oligo ARF = 8.7 × 10^−3^) (54).

In this study, we seek to expand and enhance the bioprospecting of SSAPs and the genetic tractability of *Pseudomonas* species. Through computational identification of SSAP homologs, we assembled a library of highly promising genus related SSAPs. Making use of DNA synthesis and iterative enriching recombineering, we successfully identified the most efficient performers of the library in a high-throughput manner across multiple *Pseudomonas* hosts, with the help of Oxford Nanopore Technologies long read sequencing (55,56). Notably, we discovered remarkable variants like R47, surpassing current standards in *P. putida* and *P. aeruginosa*; and R9, showcasing the most optimal configuration reported so far in *P*.*fluorescens*. Moreover, we demonstrated efficient recombineering activity in *P. taiwanensis* for the first time. These findings not only demonstrate the immense value of our high-throughput pooled recombinase screen but also significantly expand the recombineering toolboxes for the genus’ most prevalent members. Our study represents a substantial leap forward in genome editing for genetically intractable *Pseudomonas* species, offering promising avenues for transformative applications in biotechnology and beyond.

## Materials and methods

### Identification of *Pseudomonas* SSAP candidates

To comprehensively survey publicly available metagenomics databases for potential SSAP candidates, we first assembled seven SSAPs which represent the diverse nature of bacteriophage-derived recombinases with single-stranded annealing activity for multiple sequence alignment using Clustal Omega (https://www.ebi.ac.uk/Tools/msa/clustalo/). These SSAPs include redβ from *Escherichia coli*/λ phage (GeneBank: NP_040617.1), S065 from *Vibrio cholerae* (GeneBank: AAL59710.1), Plu2935 from *Photorhabdus laumondii* (GeneBank: WP_011147155.1), ERF from *Salmonella typhimurium*/P22 phage (GeneBank: NP_059596.1), Sak from *Lactococcus lactis*/ul36 phage (GeneBank: NP_663647.1), ERF from *Pseudomonas aeruginosa*/D3 phage (GeneBank: NP_061548.2) and DdRB from *Deinococcus radiodurans* (GeneBank: DAA06535.1) (Supplementary Table S1). Subsequently, we used jackHMMER (https://www.ebi.ac.uk/Tools/hmmer/search/jackhmmer) to search for protein homologs in UniProtKB within the taxonomy of Eubacteria and Viruses based on the HMM-profile built from multiple sequence alignment of the above-mentioned SSAPs. jackHMMER hits after 15 rounds were filtered for the ones relevant to *Pseudomonas* (Supplementary Table S2). Next, clustering of protein sequencing of *Pseudomonas* relevant hits was done using CD-HIT (PMID: 16731699) (http://weizhong-lab.ucsd.edu/cdhit-web-server/cgi-bin/index.cgi) with a setting of similarity = 0.7, which resulted in 48 clusters (Supplementary Table S3). By prioritizing members of each cluster based on the HMMER e-value (the smaller the e-value, the higher the homology to the SSAP HMM-profile) and the relevance to a phage source, we selected a total of 49 *Pseudomonas* SSAP candidates (Supplementary Tables S4 and S5) to screen for their recombineering activity. Another multiple sequence alignment, this time of all SSAP candidates and controls was performed using Clustal Omega in Jalview 2.11.2.7 (Supplementary Figure S1).

### Bacterial strains and media

All strains used in this research are listed in Supplementary Table S6 and were grown in Lysogeny Broth (LB) (10 g/L NaCl, 10 g/L tryptone and 5 g/L yeast extract) supplemented with the proper antibiotics (Table 1) when needed, unless stated otherwise. *E. coli* DH5α cells were made chemically competent as previously described (57) and were used for cloning purposes. Electrocompetent *Pseudomonas* strains were prepared as previously described (58) and used for recombineering experiments. After standard transformation, cells were allowed to recover in super optimal broth with catabolite repression (SOC) (31 g/L SOC powder), while Terrific Broth (TB) (12 g/L tryptone, 24 g/L yeast extract, 0.4% [v/v] glycerol, and 10% [v/v] phosphate buffer [23.12 g/L KH_2_PO_4_ and 125.4 g/L K_2_HPO_4_]) was used for the same purpose after electroporation during recombineering cycles. *E. coli* cells were grown at 37ºC and all *Pseudomonas* strains at 30ºC, even *P. aeruginosa*, whose optimal temperature is 37ºC, to prevent the activation of the thermo-inducible cIpL promoter controlling the recombinases and MutL_E36K_^PP^. Medium was supplemented with antibiotics and supplements following the concentrations shown in Table 1. *P. putida* KT2440, *P. taiwanensis* VLB129 and *P. fluorescens* SBW25 were managed in an ML-1 laboratory, whereas *P. aeruginosa* PAO1 was managed in an ML-2 under more secured conditions due to its potential pathogenicity.

**Table 1.** List of antibiotics used in this study with their corresponding concentrations for each species

Species	Kanamycin	Gentamicin	Streptomycin	Rifampicin	5-Fluorooritic acid	Nalidixic acid	Ciproflaxin
*Pseudomonas putida*	50 μg/ml	−	100 μg/ml	100 μg/ml	250 μg/ml	50 μg/ml	−
*Pseudomonas taiwanensis*	50 μg/ml	−	100 μg/ml	100 μg/ml	250 μg/ml	50 μg/ml	−
*Pseudomonas fluorescens*	100 μg/ml	−	100 μg/ml	100 μg/ml	250 μg/ml	50 μg/ml	−
*Pseudomonas aeruginosa*	−	100 μg/ml	−	100 μg/ml	−	−	0.5 μg/ml

### Plasmids

All plasmids employed during this study, as well as the oligonucleotides used to construct them can be found in Supplementary Tables S7 and S8, respectively. Putative recombinase sequences were manufactured by IDT as eBlocks of maximal 900 bps (longer sequences were divided into two separate fragments for synthesis), with BsaI compatible sticky ends. All of them were cloned into a pSEVA2514 mutL_E36K_^pp^ backbone previously amplified with primers C1 and C2 by means of Golden Gate or the Golden Gate-based SevaBrick Assembly (59). Additionally, variants R8, R9, R12, R35 and R47 were similarly cloned into a pSEVA6514 mutL_E36K_^pp^ backbone for the experiments in *P. aeruginosa* PAO1. Gibson Assembly using the NEBuilder HiFi DNA Assembly Master Mix was utilized following manufacturer’s instructions when Golden Gate did not suffice due to difficulties during the assembly process. This was the case for R26, R30, R37 and R49, which had to be amplified using the sets of primers GA R26 FW-RV, GA R30 FW-RV, GA R37 FW-RV and GA R49 FW-RV in order to be cloned into a pSEVA2514 mutL_E36K_^pp^ backbone generated with primers GA BB FW-RV. Construction of the chimeric pSEVA(2/6)514-R9-Rec2-R12-R47-PapRecT-mutLE36KPP plasmids was done via Golden Gate using aforementioned backbones pSEVA2514-mutL_E36K_^pp^ and pSEVA6514- mutL_E36K_^pp^ and DNA fragments of R9, Rec2, R12, R47 and PapRecT, amplified with primer pairs C3-C4, C5-C6, C7-C8, C9-C10, C11-C12, respectively. All resulting plasmids were transformed *via* heat shock into chemically competent *E. coli* DH5α cells for plasmid amplification and subsequently isolated using GenJET Plasmid Miniprep Kit® (Thermo Scientific). Plasmid presence was confirmed by colony PCR using Phire Hot start II DNA polymerase (Thermo Fisher Scientific) and primers V1 and V2 according to manufacturer’s guidelines. Plasmid sequence was confirmed using Sanger sequencing and primers V1 and V2 by Macrogen (MACROGEN Inc. DNA Sequencing Service; Amsterdam, The Netherlands).

### Oligonucleotides

Single-stranded (ss) DNA oligonucleotides employed in this study (Supplementary Table S8) were purchased from Integrated DNA technologies (IDT) as salt-free without further purification, resuspended in milli-Q at 100 μM and long-term stored at −20°C.

Recombineering oligonucleotides were designed according to previously optimized parameters (50,54). In brief, they were 60-mer oligonucleotides in which the mutation change was incorporated at the middle position and without phosphorothioate bonds in their sequence. They were designed complementary to the lagging strand of replicating DNA and with predicted folding energies equal to, or higher than 16 kcal/mol. Recombineering oligonucleotides used in this study were designed to confer different antibiotic resistances by means of small mutations (up to 3 nucleotides) in key genes of the different hosts: (i) *rpsL* K43T mutation in *P. putida*, *P. taiwanensis* and *P. fluorescens* conferred streptomycin resistance; (ii) *rpoB* Q518L mutation in *P. putida*, *P. taiwanensis* and *P. fluorescens* and *rpoB* D521V mutation in *P. aeruginosa* conferred rifampicin resistance; (iii) *pyrF* E50stop mutation in *P. putida*, *pyrF* Q50stop mutation in *P. taiwanensis* and *pyrF* G50stop mutation in *P. fluorescens* conferred 5-fluorooritic acid resistance; (iv) *gyrA* D87N mutation in *P. putida*, *P. taiwanensis* and *P. fluorescens* conferred nalidixic acid resistance; and (v) *gyrA* T83I mutation in *P. aeruginosa* conferred ciproflaxin resistance (Supplementary Table S8).

### High-throughput pooled recombinase screen

Recombinase library plasmids were transformed into electrocompetent *P. putida* KT2440, *P. taiwanensis* VLB120 and *P. fluorescens* SBW25 cells. Presence of the plasmids was confirmed again *via* colony PCR using Phire Hot start II DNA polymerase (Thermo Fisher Scientific) and primers V1 and V2 according to manufacturer’s guidelines. Overnight cultures of all strains containing each of the putative SSAPs and controls were diluted to OD_600_ = 0.1 and pooled into two replicas containing the control strains. Once the cultures grew to mid-log phase (OD_600_ = 0.5–0.7), samples were induced for 10 min at 42ºC, kept on ice for 5 min for inactivation of the thermo-induction, washed twice with 10 and 1 ml 300 mM sucrose, respectively, and finally resuspended in 200 μl of 300 mM sucrose. A 100 μl aliquot of competent cells was mixed with 1 μl of an appropriate 100 μM oligonucleotide for subsequent electroporation (2500 V, 200 Ω and 25 μF). Cells were allowed to recover in TB until OD = ∼0.3–0.5 before applying selective pressure *via* LB with antibiotics and overnight growth. The steps were repeated with all four oligonucleotides and then the entire four-cycle protocol was repeated by miniprepping the samples after the first round and then reintroducing the enriched library into naive host strains.

### Oxford Nanopore NGS workflow

Samples for sequencing were collected after every recombineering step and stored as 25% glycerol stocks until all cycles were completed. Samples were later grown, and plasmid content was subsequently extracted and diluted to get the optimal amount of DNA (400 ng) for the sequencing library preparation. Library preparation was done using the ONT Rapid Barcoding Sequencing kit SQK-RBK004, which fragments the DNA and adds a barcode in one step, and the ONT Flow Cell FLO-MIN106D R9 was primed using the ONT Flow Cell Priming kit EXP-FLP002, which prepares the cell for the library and provides fuel for the experiment. The Rapid Barcoding kit provided 12 barcodes, which were distributed for each one of the 11 sample-collection points (Supplementary Table S9) and for the Lambda control DNA (Control Expansion kit EXP-CTL001) provided by ONT, to ensure the libraries were of sufficient quality. Next, sequencing was initiated and monitored by the accompanying software minKNOW by ONT. Fast basecalling was chosen as our interest lies in the quantification of the library, not high accurate sequencing. Bias Voltage was adjusted when necessary, according to the guidelines on the ONT website. In between runs, the flow cell was washed (wash kit EXP-WSH004 by ONT) to ensure no traces of the previous sample remained, as we made use of the same barcodes in every library. A total of two libraries were run for each of the species.

### NGS data analysis

The raw sequencing data from the minION stored in Fast5 files was basecalled using the Guppy Basecaller (ONT) yielding FastQ files. The basecaller software allowed for demutiplexing the files based on their barcodes (1-12). Files were then merged into one FastQ file per barcode per sample. The Guppy Aligner (ONT) was used for the alignment of the merged files and generated the alignment files in BAM-format. BAM (and SAM) files were manipulated with readily available tools like Samtools, which was used in this instance to quantify the SSAP abundance over the cycles of the selective enrichment (Samtools idxstats). These data were finally used for visualizing the enrichment. The software used, minKNOW GUI and Guppy, are available on the Oxford Nanopore Technologies website and the Samtools code is freely available on Github or https://www.htslib.org/. The relevant SSAP genes of this study, R8, R9, R12, R26, R27, R35, R47, and the controls Rec2, PapRecT and λ Red, were *de novo* assembled from the raw data from barcode01 using the *flye* software version 2.8.2 (https://github.com/fenderglass/Flye), and polished with the Medaka tool to create consensus sequences and ensure the integrity of these genes in the final experiment before enrichment.

### Individual assessment of selected SSAPs

Strains containing SSAPs that had shown activity in the high-throughput pooled recombinase screen were subjected to individual assessment *via* recombineering. Recombineering cycles were performed according to the previously described standard protocol (54,60), in a similar manner than it had been done during the high-throughput pooled recombinase screen but without pooling the different samples and only for one cycle. After transformation with the corresponding oligonucleotides, cells were left to recover in 5 ml of TB at 30°C and 250 rpm. Afterwards, 15 ml of LB was added, and samples were incubated overnight under the same conditions. Next day, appropriate dilutions were cultured on to LB plates both containing and lacking the corresponding antibiotic whose resistance would have been conferred by the mutation incorporated during the recombineering cycle for subsequent efficiency calculation. Cultures without thermo-induction were included as negative controls during one of the replicas to discard any possible native or spontaneous resistance to the antibiotics. Recombineering efficiency was calculated as the ratio between antibiotic resistant and total colony forming units (CFU).

During the assay to evaluate the different recombineering efficiency at different times, cells were plated and therefore subjected to the antibiotic pressure at different times after the transformation of the oligonucleotides. Specifically, cells were plated 1, 3, 5, 7, 9 and 24 h after the addition of the TB that initiates the phase of recovery.

### Off-target analysis

In order to investigate the off-target mutations that occur in the cells during the process of recombineering when using each individual SSAP, we used the GenElute Bacterial Genomic DNA kit (Sigma-Aldrich) to isolate gDNA from two colonies of the next strains: (i) *P. putida* expressing pSEVA2514-R8-mutL, (ii) *P. putida* expressing pSEVA2514-R12-mutL, (iii) *P. putida* expressing pSEVA2514-R35-mutL, (iv) *P. putida* expressing pSEVA2514-R47-mutL, (v) *P. taiwanensis* expressing pSEVA2514-R47-mutL, (vi) *P. fluorescens* expressing pSEVA2514-R9-mutL, (vii) *P. fluorescens* expressing pSEVA2514-R47-mutL and (viii) *P. aeruginosa* expressing pSEVA6514-R47-mutL. Successful incorporation of an oligonucleotide mediating either the *rpsL* gene mutation in *P. putida*, *P. taiwanensis* and *P. fluorescens*, or the *rpoB* gene mutation in *P. aeruginosa* had been performed using one recombineering cycle in the aforementioned strains prior genomic DNA isolation. In the same way, but without undertaking any recombineering, we extracted the gDNA from one colony of each four wild-type parental strains. Isolated gDNA was sent for whole genome sequencing to Novogene Co. Ltd. (Beijing, China) for Illumina sequencing. Fastp (v0.20.0) was used to trim the raw Illumina reads for low quality and Illumina adapters. Subsequently, breseq (v0.35.5) was utilized to find the mutations using the reference genomes and annotations of *P. putida* KT2440 (NC_002947.4), *P. taiwanensis* VLB120 (NC_022738), *P. fluorescens* SBW25 (NC_012660.1) and *P. aeruginosa* PAO1 (NC_002516.2). Final number of off-target mutations was calculated as the average of the total number of non-intended mutations in the two colonies after the removal of those mutations also present in the sequencing results of the parental strains (Supplementary Table S10).

## Results

### Metagenomic discovery of genus specific SSAPs for high-throughput screening in *Pseudomonas* species

We first performed a comprehensive search of potential SSAP functional homologs in the metagenomes of *Pseudomonas* relevant bacteria species and bacteriophages. To maximize our chance to uncover active SSAPs from metagenomic databases, we manually curated a list of seven SSAPs (Supplementary Table S1) from three diverse SSAP superfamilies (RecT, Erf and Sak) with relativity high activity in prior studies to build the initial hidden Markov model (HMM) for functional homolog searching (61–63). After 15 rounds of jackHMMER searches, we identified >10^4^ hits, among which 287 come from diverse species in the *Pseudomonas* genus and 18 come from their bacteriophages (Supplementary Table S2). Protein sequence clustering of these 305 *Pseudomonas* hits resulted in 48 distinct groups (Supplementary Table S3) from which we surveyed a total of 49 *Pseudomonas* SSAP candidates (Supplementary Table S4) for gene synthesis and subsequent testing in four *Pseudomonas* species (*P*. *putida*, *P. taiwanensis*, *P*. *fluorescens* and *P*. *aeruginosa*).

The predicted 49 SSAP candidates (Supplementary Table S5) were synthesized and cloned into pSEVA2514 vectors upstream a *mutL*_E36K_^PP^ gene (the *P. putida*’s dominant mutant allele that works as a mismatch machinery repair disruptor) under the control of the inducible cI857/PL expression system (53). In addition, vectors containing the genes for Rec2 (51), PapRecT (52) and the λ Red recombineering system (64) were generated in the same fashion as controls. At this point, it is worth mentioning that our bioinformatics search yielded one candidate, R7, with a sequence that differed from that of PapRecT by only one amino acid (A201S). Furthermore, the employed λ Red recombineering system encompassed the gene encoding the SSAP Beta but also those encoding for the Exo and Gam proteins. While not required, the presence of Gam enhances ssDNA recombination efficiency in *E. coli* (35), but this effect was deemed negligible in our setup given the poor efficiencies of this system in *Pseudomonas* species (51,53).

**Figure 1.**
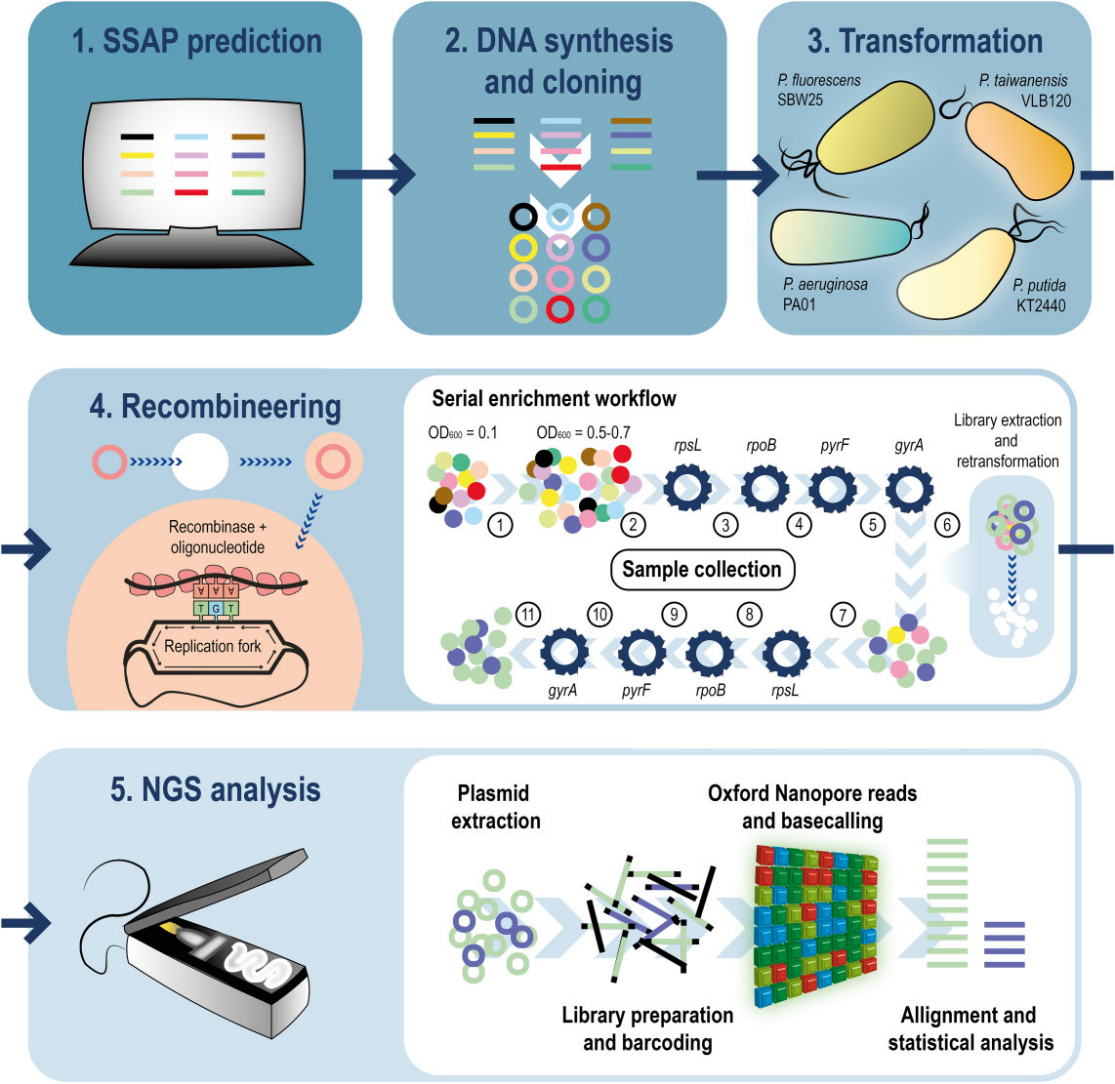
Step-by-step high-throughput pooled recombinase screen to find active SSAPs in *Pseudomonas* species. (**1**) Bioinformatics-metagenomic approach for SSAP prediction. (**2**) DNA synthesis and cloning of all the predicted SSAP candidates into pSEVA2514-*mutL*_E36K_^PP^ vectors via Golden Gate assembly. (**3**) Plasmid transformation of all the SSAP candidates into the different *Pseudomonas* species included in this study (*P. putida* KT2440, *P. taiwanensis* VLB120, *P. fluorescens* SBW25 and *P. aeruginosa* PAO1). (**4**) High-throughput pooled recombinase screen. All the strains from the same species containing the different candidates are combined in an equimolar pool of cells and grown together until reaching mid-log phase. At that point, each library of strains is exposed to four iterative recombineering cycles that mediate four specific mutations that confer different antibiotic resistances. By selecting with the appropriate antibiotics, only those cells in which the mutation has been adequately introduced will survive the pressure, enriching the pool in those cells containing active SSAPs. After the fourth mutation, all the plasmid content is extracted from the cell population for transformation of a new naïve parental strain for a second round of enrichment using the same selective mutations. Eventually, and after 8 recombineering iterations, the final population contains only those cells carrying the most efficient SSAPs. Samples were collected at the 11 different time points indicated for NGS analysis. (**5**) After extracting the plasmid content, each of the collected samples was barcoded accordingly. SSAP abundances were determined by Nanopore sequencing after basecalling and aligning the long reads to the sequence of the candidate SSAP genes.

Naïve libraries of *P. putida* KT2440, *P. taiwanensis* VLB120 and *P. fluorescens* SBW25 were then constructed to assess the activity of the SSAPs in the different hosts *via* enriching cycles of recombineering (52). For this, we used compatible oligonucleotides that introduce small mutations in the form of mismatches in the different genomes conferring specific antibiotic resistances. In this way, four assays were employed: (i) streptomycin resistance caused by a mutation in the *rpsL* gene, (ii) rifampicin resistance conferred by mutating the *rpoB* gene, (iii) 5-fluoroorotic acid (5-FOA) resistance due to the introduction of a stop codon in the *pyrF* gene, and (iv) nalidixic acid or ciprofloxacin resistance conferred after mutating the *gyrA* gene (53). After four consecutive iterations of recombineering introducing the aforementioned mutations and their corresponding selections, plasmids were extracted from the surviving cell populations and reintroduced in naïve parental strains with clean background for a second 4-assay round. Samples were collected prior to the serial enrichment process and after each of the recombineering cycles for plasmid extraction. Barcoding and library preparation using these samples was performed using the Rapid Barcoding Kit (Oxford Nanopore Technologies), which does not require amplification of the target sequence and therefore allowed us to overcome some technical challenges that could arise from using other platforms as Illumina, which could derive on biased results (52) given our primary setup and the size variability of the SSAPs. Nanopore sequencing was employed to track the presence and evolution of the different SSAP candidates during the whole screening (Figure 1). While ONT is still less accurate than other sequencing platforms (65), its purpose in this study was to help us assess the active SSAPs over the high-throughput pooled recombinase screen, not to accurately determine their sequences, which made it the best option for our objectives.

**Figure 2.**
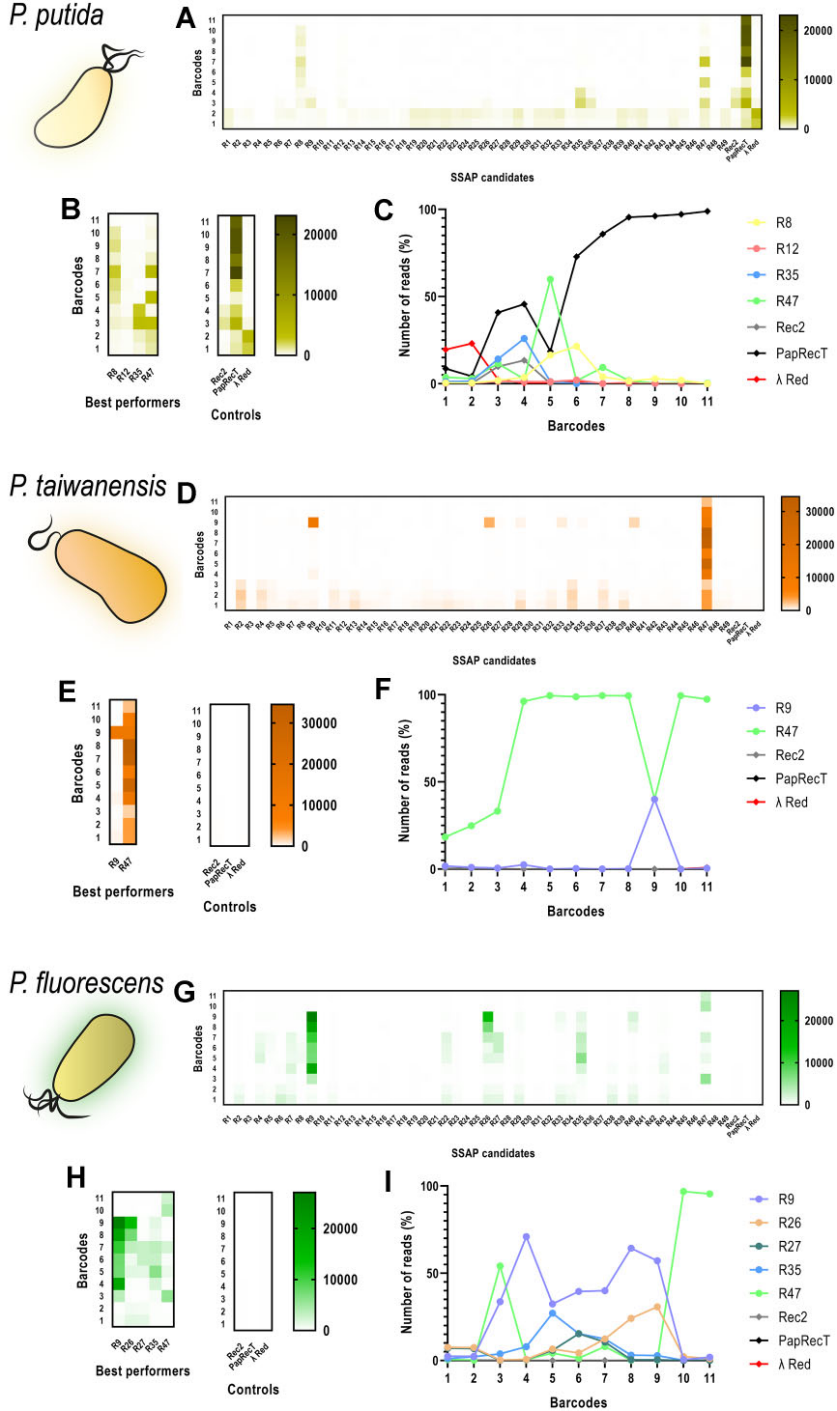
Abundance of NGS reads per SSAP over the high-throughput pooled recombinase screen cycles in *P. putida* KT2440 (yellow), *P. taiwanensis* VLB120 (orange) and *P. fluorescens* SBW25 (green). (**A**, **D** and **G**) Heatmaps depicting abundance of all 49 SSAP candidates plus controls for each of the 11 barcoded samples in *P. putida*, *P. taiwanensis* and *P. fluorescens*, respectively (mean, *n* = 2 technical). The number of reads associated to the abundance within the cell population is represented in a color scale mapping according to the depicted corresponding legends. (**B**, **E** and **H**) Heatmaps zooming into the abundance of the best performers and the three controls per species (*P. putida*, *P. taiwanensis* and *P. fluorescens*, respectively). (**C**, **F** and**I**) Percentage of reads per best performing SSAP (circles) and per control (diamonds) over the enrichment cycles in *P. putida*, *P. taiwanensis* and *P. fluorescens*, respectively. Barcodes 1 and 2 represent the samples after pooling (OD_600_ = 0.1) and after growth (OD_600_ = 0.5–0.7), prior the first recombineering cycle. Barcodes 3- 6 represent the samples after the first recombineering cycles with oligos rpsL, rpoB, pyrF and gyrA, respectively. Barcode 7 represents the samples after transformation into a naïve host. Barcodes 8–11 represent the samples after the second recombineering cycles with oligos rpsL, rpoB, pyrF and gyrA, respectively.

Application of our high-throughput pooled recombinase screen in *P. putida* KT2440 revealed the presence of four active SSAPs: R8, R12, R35 and R47 (Figure 2A–C). These four candidates were chosen based on their presence, at least up to barcode 9. Their activity (presumably related to their ARF) surpassed the one of the positive control Rec2, especially after the second recombineering cycle of the screen (barcode 4) and, while their abundance varied during the course of the enrichment, these four clearly emerged over the other candidates, which did not report any sign of recombineering activity. Furthermore, the positive control PapRecT was the most abundant SSAP of the whole library presenting an absolute number of reads significantly higher than that of the other positive control Rec2. However, its relative change in abundance did not differ much from other SSAPs over the cycles (Supplementary Figure S2). Taking into account the percentages of the cell population containing PapRecT compared to those of previous cycles, relative abundance increase of PapRecT was quite subtle after the first introduction (barcode 3) and after the subsequent reintroduction (barcode 8) of the enriched library into the naïve strain KT2440. In addition, analysis of barcodes 1 and 2, previous to the recombineering selection pressure, showed no sign of strains containing R4, R5, R11 and R46 in the initial naïve library after the pooling of samples meaning these SSAPs were finally not assessed in *P. putida* (Table 2 and Figure 2A).

**Table 2.** Absent SSAPs during the high-throughput pooled recombinase screen per species prior to the recombineering selection pressure

Species	Number of absent SSAPs in the experiment	Absent SSAPs
*P. putida*	4	R4, R5, R11, R46
*P. taiwanensis*	1	R46
*P. fluorescens*	21	R1, R3, R10, R12, R14, R17, R18, R20, R21, R24, R25, R28, R30, R32, R36, R37, R41, R44, R46, R49 and PapRecT

In *P. taiwanensis* VLB120, two active SSAPs were uncovered after the library enrichment: R9 and R47 (Figure 2D–F). R47 was the most abundant after every cycle, while R9 was mostly represented after the mutations on the *rpoB* gene (barcodes 4 and 9), likely indicating that this SSAP introduces that specific mutation more efficiently. Rec2 and PapRecT completely disappeared from the population after the first antibiotic pressure (barcode 3) rendering them seemingly inefficient in this *Pseudomonas* species, whereas λ Red showed low levels of activity. The current absence of a recombineering methodology in *P. taiwanensis* explains the lack of positive controls and the role of Rec2 and PapRecT as two additional SSAP naïve candidates during the assay in this species. Analysis of the first two barcodes revealed that the strain containing R46 was again not present during the enrichment process (Table 2 and Figure 2D).

Five active SSAPs were identified in *P. fluorescens* SBW25. R9 was the most represented candidate during all the cycles, but R26, R27, R35 and R47 reported activity as well with variable numbers of reads throughout the enrichment process (Figure 2G–I). Once again, the controls Rec2 and λ Red showed very low ARF levels, while PapRecT was not even detected before the selection pressure. Unfortunately, a large amount of SSAP candidates were not detected in barcodes 1 and 2, with 21 strains missing the assessment in *P. fluorescens* (Table 2 and Figure 2G). This species’ ability to readily acquire resistance to antibiotics might have caused the strains to lose their SSAP plasmids as they became resistant to kanamycin. This evolved advantage against the antibiotic most likely resulted in all these strains not needing their plasmids for survival (66).

### New SSAPs prove to efficiently mediate recombineering

Once that the high-throughput pooled recombinase screen yielded active variants in the different species, SSAPs were assessed individually to measure ARF at a clonal level. Since editing efficiency is typically dependent of the target locus (53,54), we measured ARF at two different genomic loci: *P. putida rpsL* and *pyrF* genes, *P. taiwanensis rpsL* and *rpoB* genes, *P. fluorescens rpsL* and *pyrF* genes, and *P. aeruginosa rpoB* and *gyrA* genes. The *rpsL* locus was selected for the first three species to provide some degree of comparability among the different *Pseudomonas*. The choice of the second locus was arbitrary in an attempt to include at least once all the mutations that confer antibiotic resistance used in this study. For *P. aeruginosa*, the *rpoB* and *gyrA* loci were chosen for a better comparison with similar tests in existing literature (52). For an easy screening, we made use again of oligonucleotides that confer different antibiotic resistances through small mutations as we had previously done during our high-throughput pooled recombinase screen (Figure 1).

**Figure 3.**
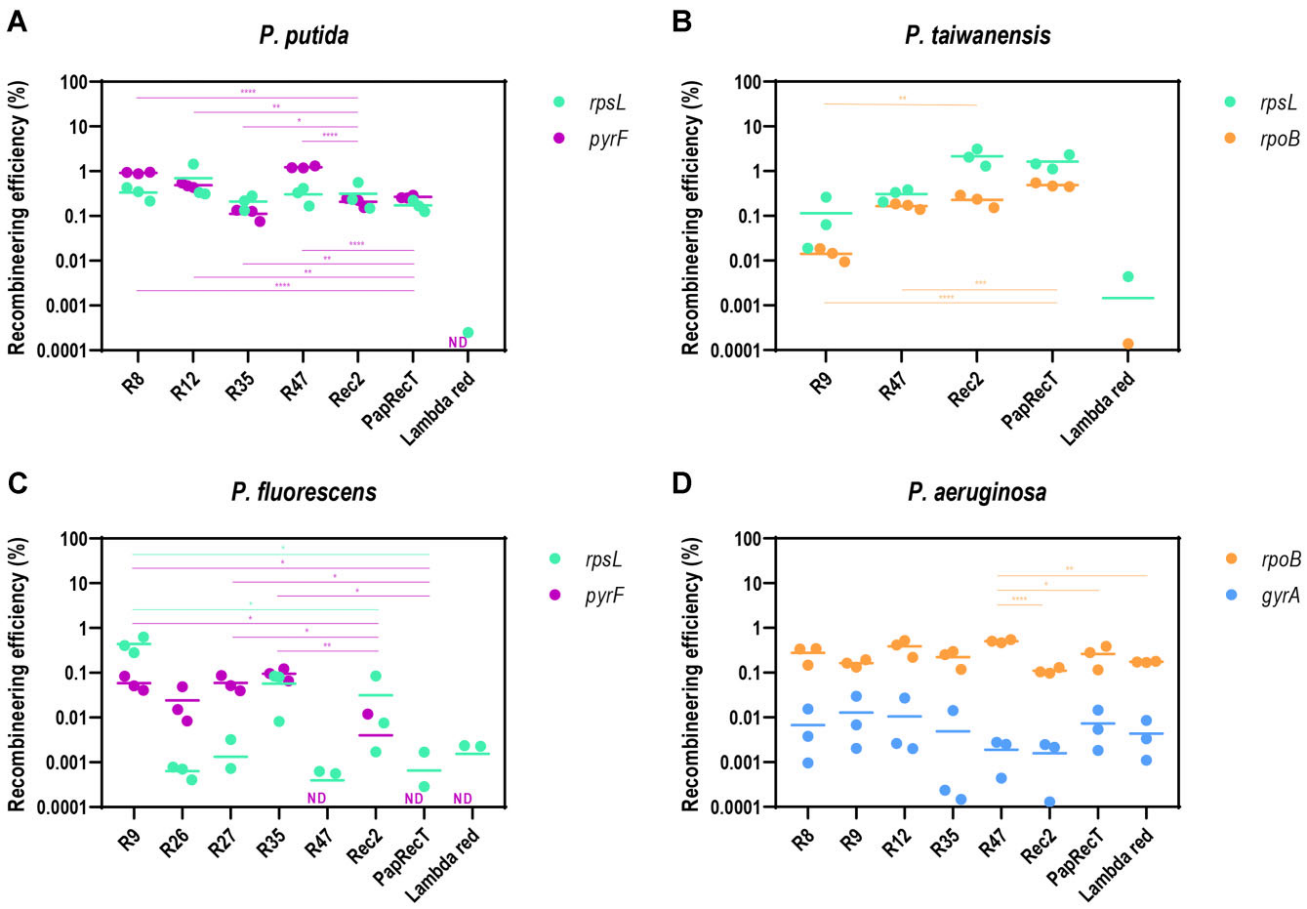
Recombineering efficiency of selected SSAPs in individual strains in percentage. Graphs display recombineering efficiency mediating: (**A**) *rpsL* K43T (green) and *pyrF* E50stop (purple) mutations in *P. putida* KT2440, (**B**) *rpsL* K43T (green) and *rpoB* Q518L (orange) mutations in *P. taiwanensis* VLB120, (**C**) *rpsL* K43T (green) and *pyrF* G50stop (purple) mutations in *P. fluorescens* SBW25, and (**D**) *rpoB* D521V (orange) and *gyrA* T83I (blue) mutations in *P. aeruginosa* PAO1. Recombineering efficiency was calculated as the ratio between antibiotic resistant CFUs growing in pressure plates of LB with the corresponding antibiotic and total CFUs growing in LB plates. ND labels indicate that no mutants were detected under those specific circumstances of mutation and SSAP. Non-induced samples were included as controls and were subtracted from the absolute values. Only significant values are indicated for a parametric two-tailed *t-*test between two groups, where *P ≤ 0.05, **P < 0.01, ***P < 0.001 and ****P < 0.0001; non-significant values were not depicted (mean ± s.d., *n* = 3 biological).

In *P. putida*, the results from the controls Rec2, PapRecT and λ Red were consistent with those obtained in previous research (51,54). While the number of reads of PapRecT in the pooled population of the high-throughput screen was significantly higher than those of Rec2 or of any of the candidates, its individual assessment (0.17 ± 0.05% for *rpsL* and 0.26 ± 0.01% for *pyrF*) resulted in ARFs in the same range as those obtained with Rec2 (0.31 ± 0.21% for *rpsL* and 0.20 ± 0.04% for *pyrF*). On the other hand, λ Red showed low recombination efficiency (0.00 ± 0.00% for both *rpsL* and *pyrF*), which is in agreement with the results of the HT SSAP screening and prior literature (51). Regarding the selected SSAP candidates, R8 yielded ARFs of 0.33 ± 0.10% for *rpsL* and 0.92 ± 0.04% for *pyrF*. R12 scored 0.48 ± 0.05% for *pyrF* and an inconsistent 0.69% with a standard deviation of 0.64 for *rpsL*. R35 showed the lowest efficiencies of the individual assessment with 0.21 ± 0.07% for *rpsL* and 0.11 ± 0.03% for *pyrF*. Lastly, R47 reported the highest levels for *pyrF* with a 1.22 ± 0.07%, and an ARF of 0.30 ± 0.12% for *rpsL* (Figure 3A). While the individual performance of these four candidates was found within the same range than the ones of Rec2 and PapRecT for the *rpsL* mutation (not significative differences according to our parametric two-tailed *t*-test), in the case of *pyrF*, R8, R12 and R47 performed significantly better than both positive controls.

Rec2 and PapRecT showed the best individual results in *P. taiwanensis* despite their scarce presence in the high-throughput pooled recombinase screen of this bacterium (2.15 ± 0.90% and 1.63 ± 0.62% for *rpsL*, and 0.22 ± 0.06% and 0.48 ± 0.04% for *rpoB*, respectively) (Figure 3B). In contrast, R47, which was the most represented candidate of the pooled library over the enrichment cycles, yielded lower ARFs (0.30 ± 0.09% for *rpsL* and 0.16 ± 0.02% for *rpoB*). However, significative differences were only observed between the results of R47 and PapRecT for the *rpoB* mutation, which can most likely be explained due to the variance of the Rec2 and PapRecT replicates. Lastly, R9 showed low ARF levels (0.11 ± 0.12% for *rpsL* and 0.01 ± 0.004% for *rpoB*) while no ARF was detected at all for the λ Red system in this *Pseudomonas* species either.

The individual assessment of SSAPs in *P. fluorescens* resulted in R9 as the best SSAP with an ARF of 0.43 ± 0.17% for *rpsL*, which was significantly higher than any other candidate, and an ARF of 0.05 ± 0.02% for *pyrF* (Figure 3C). R26, R27 and R35 yielded similarly low results for the *pyrF* mutation (0.02 ± 0.02%, 0.05 ± 0.02% and 0.09 ± 0.02%, respectively). However, of these three, only R35 could efficiently mediate the *rpsL* mutation (0.05 ± 0.04%) while R26 and R27 showed insignificant ARF levels <0.002%. This was also the case for R47, PapRecT and the λ Red system, which did not score ARFs >0.002% for neither of the mutations. Rec2 did nonetheless showed low ARFs: 0.03 ± 0.04% for *rpsL* and 0.004 ± 0.006% for *pyrF*.

Finally, we evaluated the most efficient recombinases from the three previous *Pseudomonas* in the related species *P. aeruginosa* PAO1 (Figure 3D). Given the natural resistance of this species to Kanamycin (67), selected SSAPs were cloned into pSEVA6514 plasmids that confer resistance to gentamicin. For the test, we also utilized two different antibiotic resistance readouts, the *rpoB* D521V and the *gyrA* T83I mutations, which had been previously characterized in this bacterium (52). In general, all our candidates, namely R8, R9, R12, R35, R47, Rec2, PapRecT and the λ Red system, showed recombineering activity in *P. aeruginosa*, with higher ARF levels for the *rpoB* than for the *gyrA* mutation, which were all ≤0.01%. Our *rpoB* results of PapRecT and Rec2 (0.26 ± 0.13% and 0.11 ± 0.01%, respectively) were consistent with those obtained in previous research (52), *i.e*., Rec2 seems to have some activity in this species but it is less suitable for editing in *P. aeruginosa* than PapRecT. In contrast, when we tested the λ Red system, we obtained a relatively modest ARF of 0.17 ± 0.005%, which was higher than the reported ARFs by other previous studies (52,68,69). Furthermore, all R8 (0.27 ± 0.11%), R9 (0.16 ± 0.03%), R12 (0.38 ± 0.15%) and R35 (0.22 ± 0.09%) yielded ARFs in the same range as PapRecT, while R47 significantly surpassed all the other candidates and controls with a notable ARF of 0.50 ± 0.04%.

### Allelic replacement rate differs across SSAPs

Motivated by the differences between the behaviour of the SSAPs within the pooled library and in the individual tests, we performed an assay to determine if other intrinsic characteristics beyond the ARF could have influenced the results of the high-throughput pooled recombinase screen. Our hypothesis was that SSAPs could have different action rates and that faster candidates would take over naïve cell populations that were quickly subjected to the pressure thanks to a faster incorporation of the resistance mutation. In this context, action rate could be determined by the rate of protein production, the binding affinity between the SSAP and the DNA oligonucleotide, and the catalytic activity of the SSAP to anneal this substrate to the lagging strand genomic template between the Okazaki fragments. During our high-throughput pooled recombinase screen, we applied the selection pressure to our samples via antibiotic addition around 4 h after the end of the recombineering cycle (when OD_600_ reached 0.3–0.5). At that moment, the naïve cell population likely contained more resistant cells mutated with the faster SSAPs causing an increase on the percentage of those cells, which would not be derived from a higher efficiency but from a higher rate. To test our hypothesis, we compared the recombineering efficiency of PapRecT and R12 in *P. putida* over time using the readout of the *rpsL* K43T mutation. Previously, PapRecT had shown a significantly higher number of reads than R12 during the high-throughput pooled recombinase screen: 98.96% versus 2.24%. This phenomenon was clearly visible since the first recombineering cycle (barcode 3). However, when tested separately, R12 surpassed the ARF of PapRecT.

**Figure 4.**
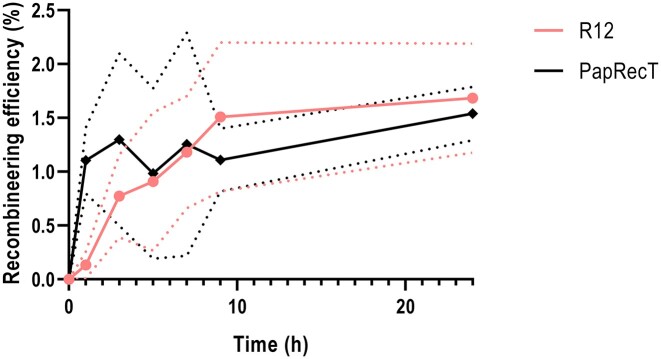
Comparative of the editing efficiencies of R12 and PapRecT when subjected to pressure at different time points after recombineering during 24 h. Full lines represent average values while dashed lines represent the standard deviation field. Graph displays recombineering efficiency mediating the *rpsL* K43T mutation in *P. putida* KT2440 which was calculated as the ratio between streptomycin resistant CFUs growing in LB-sm and total CFUs growing in LB plates. Samples before the recombineering cycle were included as controls and were subtracted from the absolute values (mean ± s.d., *n* = 3 biological)

In this experiment, we subjected to pressure two different cell populations containing the aforementioned SSAPs at different times after the recombineering cycle. R12 yielded again a higher final average ARF than PapRecT (1.68 ± 0.50% versus 1.53 ± 0.24%) even though their differences were not significant after 24 h (Figure 4). Nevertheless, it was at the early stages of the assay when we could observe some differences. Interestingly, PapRecT reached 71.79% of its final ARF (1.10 ± 0.30%) only 1 h after the end of the recombineering cycle whereas R12 needed >7 h to reach an equivalent. Protocols of cyclic recombineering usually recommend an incubation of at least 3 h before proceeding with a consecutive cycle (stemming from *E. coli*’s doubling time) (70) or reaching an OD_600_ of mid-log phase (guaranteeing at least two replications) (53,70) to ensure that the introduced mutation is inherited across the population. In the case of PapRecT, only one hour was needed to almost achieve the target population of mutants, shedding light on the overwhelming cell population percentages containing this recombinase during the enrichment process (Figure 2A–C).

### Combining several SSAPs does not result in a synergetic effect

In an attempt to enhance recombineering efficiencies, we combined five of our best SSAPs (R9, R12, R47, Rec2 and PapRecT) on a single chimeric pSEVA(2/6)514 mutL_E36K_^PP^ plasmid under the control of the inducible cI857/PL expression system (71). Previous studies had demonstrated that increasing the gene expression of the SSAP by using strong ribosomal binding sites (RBS) could result in a significant improvement of editing efficiency (52). In this regard, we hypothesized that multiple SSAP variants expressed simultaneously could favour allelic replacement as a consequence of their synergetic effect. To assess the efficiency of the chimeric plasmid in the different *Pseudomonas*, we targeted the *rpsL* (in *P. putida*, *P. taiwanensis* and *P. fluorescens*) and the *rpoB* (in *P. aeruginosa*) genes to measure ARF.

**Figure 5.**
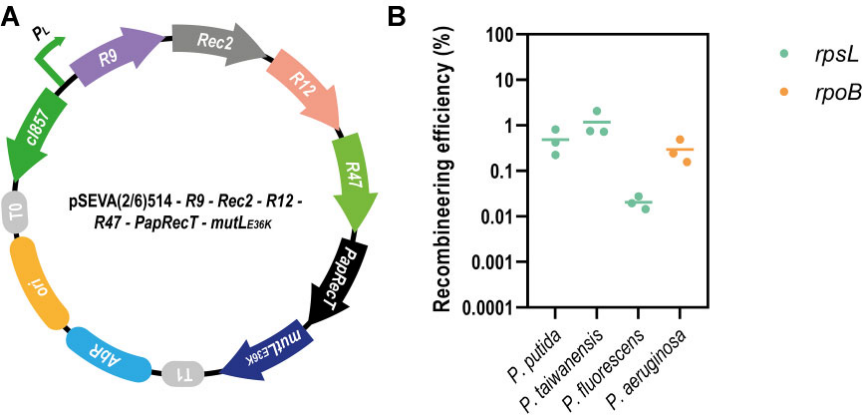
Recombineering efficiency of a chimeric plasmid encompassing five active SSAPs. (**A**) The chimeric pSEVA(2/6)514-*R9-Rec2-R12-R47-PapRecT-mutL_E36K_* plasmid, harboring the thermolabile cI857 repressor (dark green) controlling a synthetic operon of recombinase genes. Antibiotic resistance gene (blue) varied between kanamycin in pSEVA2514, which was used for *P. putida*, *P. taiwanensis* and *P. fluorescens*; and gentamicin in pSEVA6514, which was used for *P. aeruginosa*. The origin of replication RSF1010 (#5 according to the SEVA nomenclature) is depicted in yellow, and the rho-independent transcriptional terminators of phage lambda and the rrnB operon of *E. coli* (T0 and T1, respectively, according to the SEVA nomenclature) are depicted in gray. (**B**) Editing efficiencies of the chimeric pSEVA(2/6)514-*rec2*-*r9*-*r12*-*r47*-*paprecT*-*mutL*_E36K_^PP^ plasmid in individual strains in percentage. Graphs display recombineering efficiency mediating: *rpsL* K43T mutation (green) in *P. putida* KT2440, *rpsL* K43T mutation (green) in *P. taiwanensis* VLB120, *rpsL* K43T mutation (green) in *P. fluorescens* SBW25, and *rpoB* D521V (orange) mutation in *P. aeruginosa* PAO1. Recombineering efficiency was calculated as the ratio between antibiotic resistant CFUs growing in pressure plates of LB with the corresponding antibiotic and total CFUs growing in LB plates (mean ± s.d., *n* = 3 biological).

No impaired growth was observed during cell cultivation, and expression of the multiple SSAP genes, alongside *mutL*_E36K_^PP^, was only induced at the beginning of the recombineering cycle. In this experiment, the percentage of mutated cells was 0.48 ± 0.30% in *P. putida*, 1.18 ± 0.77% in *P. taiwanensis*, 0.02 ± 0.006% in *P. fluorescens* and 0.29 ± 0.17% in *P. aeruginosa* (Figure 5). In none of the cases, the efficiency achieved by the chimeric plasmid reached the average obtained with the best species-specific SSAP individually. However, these differences were not significant suggesting that the best candidates (R12 for *P. putida*, Rec2 for *P. taiwanensis* and R47 for *P. aeruginosa*) still performed equally well as part of the recombinase cassette, except in the case of *P. fluorescens*, where R9 achieved a 0.43 ± 0.17% efficiency for *rpsL* when expressed individually but only a 0.02 ± 0.006% when expressed in the chimeric plasmid.

### Off-target effect of active SSAPs

To obtain an even more comprehensive view of the SSAPs identified in this study, we examined the accumulation of off-target mutations using whole genome sequencing data from different strains that had gone through one successful recombineering cycle. SSAPs were cloned in the same pSEVA(2/6)514 mutL_E36K_^PP^ vectors meaning that the mismatch machinery repair disruptor gene was expressed alongside the SSAP gene upon 42ºC induction. We studied the off-target effect of the most efficient variants in *P. putida* (R8, R12 and R35) and *P. fluorescens* (R9). In addition, we studied the off-target effect of the variant R47 in the four different *Pseudomonas* hosts. *P. putida* cells producing R8 and R12 showed an elevated number of off-targets mutations (14 and 11.5, respectively), whereas *P. putida* cells producing R35 and *P. fluorescens* cells producing R9 resulted in a lower off-target effect, with 2 and 1 undesired mutations each (Table 3 and Supplementary Table S10). When looking at the results of cells expressing the gene for R47, we observed inconsistencies across species. *P. putida* and *P. taiwanensis* cells producing this variant contained high numbers of mutations (13.5 and 15.5, respectively), which were within the same range than those shown by R8- and R12-producing *P. putida* cells. However, *P. fluorescens* and *P. aeruginosa* cells carrying R47 exhibited lower numbers, 1 and 3.5 off-target mutations, which were comparable to those obtained for R9 in *P. fluorescens* and R35 in *P. putida* (Table 3 and Supplementary Table S10).

**Table 3.** Off-target mutations per colony after one recombineering cycle (*rpsL* gene mutation in *P. putida*, *P. taiwanensis* and *P. fluorescens*, and *rpoB* gene mutation in *P. aeruginosa*)

Plasmid	Species	*N*	Off-target mutations
pSEVA2514-R8-mutL_E36K_^PP^	*P. putida*	2	14 ± 5.66
pSEVA2514-R12-mutL_E36K_^PP^	*P. putida*	2	11.5 ± 3.54
pSEVA2514-R35-mutL_E36K_^PP^	*P. putida*	2	2 ± 1.41
pSEVA2514-R47-mutL_E36K_^PP^	*P. putida*	2	13.5 ± 2.12
pSEVA2514-R47-mutL_E36K_^PP^	*P. taiwanensis*	2	15.5 ± 7.78
pSEVA2514-R9-mutL_E36K_^PP^	*P. fluorescens*	2	1 ± 0.00
pSEVA2514-R47-mutL_E36K_^PP^	*P. fluorescens*	2	1 ± 0.00
pSEVA6514-R47-mutL_E36K_^PP^	*P. aeruginosa*	2	3.5 ± 3.54

## Discussion

In this study, we screened a library of SSAP candidates for the genus *Pseudomonas* in four representative species utilizing a user-friendly, streamlined, cost-effective and high-throughput pooled recombinase screen. We pursued a five-step strategy: (1) a bioinformatic-metagenomic approach for candidate prediction; (2) DNA synthesis and cloning of the most relevant candidates into a library of vectors; (3) transformation into the *Pseudomonas* systems; (4) a high-throughput pooled recombinase screen to enrich the best variants within the library; and (5) a final deep sequencing step with the minION Mk1B and the Rapid Barcoding Sequencing kit by ONT (Figure 1). Using this strategy, we identified a number of active variants for the studied species which were later characterized individually. Four active SSAPs, R8, R12, R35 and R47 were uncovered for *P. putida* KT2440. While their efficiency was, in general, in the same range than previous standards Rec2 and PapRecT, our candidates R8, R12 and specially R47 surpassed the controls’ ARF levels when targeting specific loci. In *P. taiwanensis* VLB120, we identified two new active SSAPs, R9 and R47, and confirmed that *P. putida*’s controls Rec2 and PapRecT also have recombineering activity in this species. The latter two showed outstanding efficiencies in *P. taiwanensis*, almost one order of magnitude higher than those obtained in *P. putida* suggesting that these variants are preferred for recombineering in this species. Six SSAPs showed some activity in *P. fluorescens* SBW25, R9, R26, R27, R35, R47 and Rec2, but only R9 yielded significant ARF levels when tested individually. While previous *P. putida* standards did not work in *P. fluorescens* as efficiently as they did in *P. taiwanensis*, newly characterized R9 represents a promising option for fostering recombineering applications in this species. Lastly, a set of the most active candidates (R8, R9, R12, R35, R47, Rec2, PapRecT and λ Red) was tested in *P. aeruginosa* PAO1. All of them showed recombineering activity in this species, but R47 was highlighted as the best performer, even surpassing the paradigm PapRecT in the conditions we tested.

To ensure that we were taking into account the natural diversity of SSAPs in candidate prediction, we carefully selected seven representative SSAPs based on their representative occupying positions in the three SSAP superfamilies and their relatively well-characterized recombineering activity. Due to the cost limitation of gene synthesis and capability of simultaneous testing of many candidates, we devised a set of simple yet efficient filtering steps to help prune the initial library to a modest-size collection. The fact that we successfully harvested half a dozen new SSAPs with significant recombineering activity in four different *Pseudomonas* species from a relatively small number of candidates suggests that our choice of this set of broad-range ‘founder’ sequences coupling with subsequent genus-specific filtering is efficient for enriching functional relevant SSAP homologs. This implies that such metagenomic bioinformatics workflow may be applicable for predicting active SSAPs in other bacterial genus.

We chose the MinION Mk1B by Oxford Nanopore Technologies for NGS analysis due to its time and cost efficiency, overcoming technical challenges compared to Illumina (72). ONT’s simpler library preparation allows in-house real-time sequencing (56,73,74). Though less accurate than Illumina (65), it served our objective of assessing active SSAPs in high-throughput, favoring data quantification over sequence accuracy.

Analysing the results of the enrichment of the naïve library in *P. putida*, it is notable that the control PapRecT seemed to be significantly more present than any other candidate (including the highly similar R7) or control (Figure 2A–C). This SSAP’s efficiency had previously been shown to be within the same range than the control Rec2 (54), which was later confirmed by our individual assessment of recombinases. Despite this inconsistency, the abundance of PapRecT remained relatively stable over the selective cycles (Supplementary Figure S2), which could indicate that once that an efficient SSAP becomes the most prominent member of the cluster, it will likely take over the whole population. This dominance effect could have masked the performance of other SSAPs, highlighting an important drawback of this kind of high-throughput screening methods, where the different elements are competing for their place within the cell population. Individual testing of the active variants and the controls revealed their efficiencies in a non-competitive environment. As mentioned, PapRecT exhibited then ARF levels consistent with those obtained in previous studies (Figure 3A), but this was not the only inconsistency that was observed between the high-throughput pooled recombinase screen results and the individual assessment. R12, despite being the one with the lowest numbers out of the four active candidates in the pooled recombinase screen of *P. putida* (Figure 2B), ended up showing similar levels of recombineering to the other active candidates and positive controls (Figure 3A), an effect that was probably caused by PapRecT dominating the population. Similarly, Rec2 and PapRecT reached very high ARF levels during their individual evaluation in *P. taiwanensis* (Figure 3B) whereas they had been completely overshadowed by R47 earlier during the high-throughput pooled recombinase screen (Figure 2E).

Differences between the pooled and the individual assessment raise the question whether there could be other factors influencing the performance of a SSAP beyond its intrinsic recombineering efficiency, such as the action rate. We confirmed this hypothesis by comparing the ARF reached by PapRecT and R12 at different time points after recombineering. After only 1 h, the former had reached >70% of the efficiency that it would reach after one day, while R12 would need six more hours to achieve these levels, demonstrating that different SSAPs mediate mutations at different rates (Figure 4). On the one hand, specific protein toxicity could affect the growth rate of cells carrying particular variants. This phenomenon has previously been described for Rec2, whose toxicity might have led to a longer lag phase or higher death rate of Rec2-expressing cells after transformation (51). On the other hand, alongside toxicity affecting growth rate, other aspects such as the process of homology detection and annealing by the SSAP might determine their action rate. For the λ Red system, it has been described that a complex needs to be formed between two redβ proteins bound to the ssDNA (Δ*G*_dimerization_) as well as hybridization between the matching strands (ΔG_hybridization_) in order to overcome the energetic barrier which inhibits interaction of the ssDNA bound to redβ with the host DNA (ΔG^‡^). The energy required for this interaction, or clamping, which is larger than the thermal energy (Boltzmann constant (*k*_B_) × absolute temperature [*T*]) can only be generated when the sequence homology is adequate (75). The rate at which these processes occur can differ and might explain some of the inconsistencies between the two assays.

Annealing of phage RecT proteins has been linked to specific interactions with bacterial single stranded binding proteins (SSBs), which mainly relies on the recognition of seven amino acids at the SSB’s C-terminal. This interaction appears to be host-specific, hence the host tropism displayed by λ redβ for disparate bacterial species. However, some RecT proteins, like PapRecT, can function more broadly by interacting with multiple SSBs (49), whose portability seems to rely on the conservation of the C-terminal sequence of the SSBs. This sequence is conserved among the different *Pseudomonas* of this study. Specifically, all of them shared the last nine amino acids at the SSB’s C-terminal (DSFDDDIPF), out of which, the last seven are also shared with *E. coli*. From the newly found SSAPs, R47 postulates as a broadly acting variant given its activity in all four tested species. This candidate derives from a phage of *Pseudomonas duriflava*, a close relative of the tested *Pseudomonas* species (Table 4), to which the conservation of the last nine amino acids of the SSB’s C-terminal also extends. While the association between ARF and taxonomic proximity has often been questioned in previous literature (51,52,62), seeking SSAPs in taxonomically related species has also been successful in numerous occasions (48,76–78). In our study, among our best performers, only R12 was distinctly derived from one of the tested species (four of them were derived from yet unclassified *Pseudomonas* species) (Table 4). Further investigation into host specificity in different bacterial species might provide further insights into this question.

**Table 4.** Overview of the origin of the best performing SSAPS in the high-throughput pooled recombinase screen of the different *Pseudomonas* species

Recombinase	GenBank description	Original species
R8	DNA recombinase	*Pseudomonas* sp. strain M1
R9	Phage recombination protein β	*Pseudomonas* sp. 91RF
R12	Phage recombination protein β	*Pseudomonas putida*
R26	Uncharacterized	*Pseudomonas* sp. AU12215
R27	Recombination protein recT	*Pseudomonas psychrotolerans*
R35	DNA recombinase	*Pseudomonas* sp. ICMP 8385
R47	Phage recombination protein β	*Pseudomonas duriflava*

In addition to the limitation of the study related to ‘slow’ potential active SSAPs masked by faster candidates, we encountered the problem of missing variants after the pooling of samples during the enrichment processes (Figure 2A, D and G). Especially for *P. fluorescens*, a lot of candidates (21) were not present during the high-throughput pooled recombinase screen after pooling all strains. This species’ ability to readily acquire resistance to antibiotics might have caused the strains to lose their recombinase plasmids as they became resistant to kanamycin themselves and the presence of their plasmids obsolete for survival (66). It is probable that the development of resistance to the antibiotic during the cloning steps led to both plasmid loss and a growth defect in these strains, as illustrated in Supplementary Figure S3. This observation occurred subsequent to the execution of the high-throughput pooled recombinase screen, which explains why we did not employ gentamicin vectors for *P. fluorescens*, in contrast to the approach adopted for *P. aeruginosa*. Moreover, R46 was found to be absent in all three experiments of enrichment, which likely indicates that this variant presents a high toxicity for all the strains, similar to what was described for Rec2 (51) but in a much higher level of lethality. In this regard, to guarantee that the potential effects of different SSAPs on the cell did not influence the outcomes of our experiments, we ensured that the different strains of the same species exhibited comparable growth rates and final OD_600_ values. In cases where this pattern was not observed, it was found that those particular strains contained SSAPs that were later determined to be absent during the high-throughput pooled recombinase screen prior to the recombineering selection pressure (Supplementary Figure S3). Another major drawback is that ARF appears to be affected by the target locus and consequently the oligonucleotide sequence, which results for some SSAPs showing different outcomes depending on the target gene. Thus, the design of the oligonucleotides influences recombineering efficiency as secondary structures can occur for oligonucleotides that target regions containing high GC content or repetitive sequences, hence decreasing efficiency (40). For example, we observe that R9 is more efficient when targeting *rpoB* (barcodes 4 and 9) both in *P. taiwanensis* and *P. fluorescens* (Figure 2F and I).

As efficiency appears to be dependent on the targeted genomic region and also the action rate of the SSAP, a result observed in similar studies in other organisms (40,46,47), we attempted to synergize the activity of several of the best variants by combining them in a single plasmid. In addition to providing higher ARF levels, this chimeric plasmid could be introduced in any *Pseudomonas* host regardless which mutation needed to be incorporated, skipping the selection of the SSAP best suited for the situation. Unfortunately, this synergetic effect was not observed in any of the species, concluding that most likely the levels of SSAP protein are not the limiting factors of the allelic replacement process as it had been previously suggested (54). The chimeric plasmid however was able to report similar efficiencies to those obtained by the best performers individually in the different *Pseudomonas*, except *P. fluorescens*, which still makes it a useful tool to use indiscriminately for different species (Figure 5).

Finally, we assessed the accumulation of off-target mutations while using the newly characterized SSAPs (Table 3). Cells expressing R9 and R35 showed off-target results within the same range than data previously reported for λ Red, PapRecT and Rec2 (1.0 ± 0.7, 3.3 ± 0.6, and 1.7, respectively) (52,54). This was also the case for R47-expressing *P. fluorescens* and *P. aeruginosa* cells. Unfortunately, *P. putida* and *P. taiwanensis* cells expressing the same SSAPs accumulated elevated numbers of mutations. These differences on the off-target effect of R47 in the four *Pseudomonas* species could be due to the mismatch repair (MMR) machinery disruptor employed in all the plasmids of this study, MutL_E36K_^PP^, which is the dominant-negative mutant of *P. putida* KT2440 MutL. While the MutL proteins of *P. putida* and *P. taiwanensis* share a 98.6% of identity, the one of *P. putida* shares with the ones of *P. fluorescens* and *P. aeruginosa* only 85.5 and 81.7%, respectively. This could indicate that the transient expression of MutL_E36K_^PP^ could have not disabled MMR completely or nothing at all in the latter two species, which would have resulted in the observed reduced amount of off-target mutations. Even though previous studies have demonstrated that this transient overexpression of dominant-negative MutL mutants greatly reduces the off-target effect when compared to complete inactivation of the MMR machinery (41,60), we observed an elevated accumulation of this type of mutations only in those species in which MutL_E36K_^PP^ had most likely taken effect. *P. putida* cells expressing the other two SSAPs, R8 and R12, also showed higher background mutation rates, which would support this hypothesis. R35, conversely, could have not yielded the same results due to its lower overall efficiency in this host. Given that the off-target effects are one of the biggest constrains of recombineering (34), this phenomenon deserves further investigation focusing on SSAP specificity and MMR inhibition.

Overall, this study provides new means for efficient recombineering in four *Pseudomonas* species, one of them previously missing any tool for this kind of genome editing. In two cases, the barrier of the 1% ARF was overcome (34), with R47 in *P. putida* KT2440 and both with Rec2 and PapRecT in *P. taiwanensis* VLB120, while values >0.4% ARF were achieved for *P. fluorescens* and *P. aeruginosa* with R9 and R47, respectively. These findings hold the promise of significantly expanding the scope and throughput of genetic manipulation of these organisms, thereby enabling more complex functional genomic analyses and increasing their relevance as a biotechnological chassis. Furthermore, this work contributes to a better understanding of SSAP performance during the recombineering process and improving SSAP screening methods. The fact that 49 SSAPs yielded several efficient candidates provides confidence in the methodology to find effective SSAPs for *Pseudomonas* sp. and other bacterial species.

## Supplementary Material

gkad1024_Supplemental_Files

## Data Availability

The NGS datasets underlying this article (ONP high-throughput pooled recombinase screen and Illumina whole-genome sequencing for off-target analysis) are available in the European Nucleotide Archive (ENA) at https://www.ebi.ac.uk/ena/browser/homeunder under the accession number PRJEB56403.

## Notes

Present address: Zhuobin Liang, Institute of Molecular Physiology, Shenzhen Bay Laboratory, Shenzhen 518132, China.
