# Supplementary material for: Metagenomics harvested genus-specific single-stranded DNA-annealing proteins improve and expand recombineering in Pseudomonas species
Source: Nucleic Acids Res. 2023 Nov 6;51(22):12522–36. doi: 10.1093/nar/gkad1024 (PMC10711431; doi:10.1093/nar/gkad1024)
Supplement: gkad1024_Supplemental_Files [file gkad1024_supplemental_files.zip › Supplementary Figures - Metagenomics harvested genus-specific single-stranded DNA-annealing proteins improve and expand recombineering in Pseudomonas species.pdf]

Supplementary file 1

Metagenomics harvested genus-specific single-stranded DNA-annealing proteins improve and expand recombineering in *Pseudomonas* species

Enrique Asin-Garcia[1,2], Luis Garcia-Morales[1], Tessa Bartholet[1], Zhuobin Liang[3,4,5], Farren J. Isaacs[3,4], Vitor A. P. Martins dos Santos[1,2,*]

[1] Laboratory of Systems and Synthetic Biology, Wageningen University & Research, Wageningen, 6708 WE, The Netherlands

[2] Bioprocess Engineering Group, Wageningen University & Research, Wageningen, 6700 AA, The Netherlands

[3] Department of Molecular, Cellular, and Developmental Biology, Yale University, New Haven, CT 06520, USA

[4] Systems Biology Institute, Yale University, West Haven, CT 06516, USA

[5] Institute of Molecular Physiology, Shenzhen Bay Laboratory, Shenzhen 518132, China

[6] LifeGlimmer GmbH, Berlin, 12163, Germany

* To whom correspondence should be addressed. Tel: +31317482865; Email: vitor.martinsdossantos@wur.nl

Supplementary Figure S1. Multiple sequence alignment of the SSAP candidates.

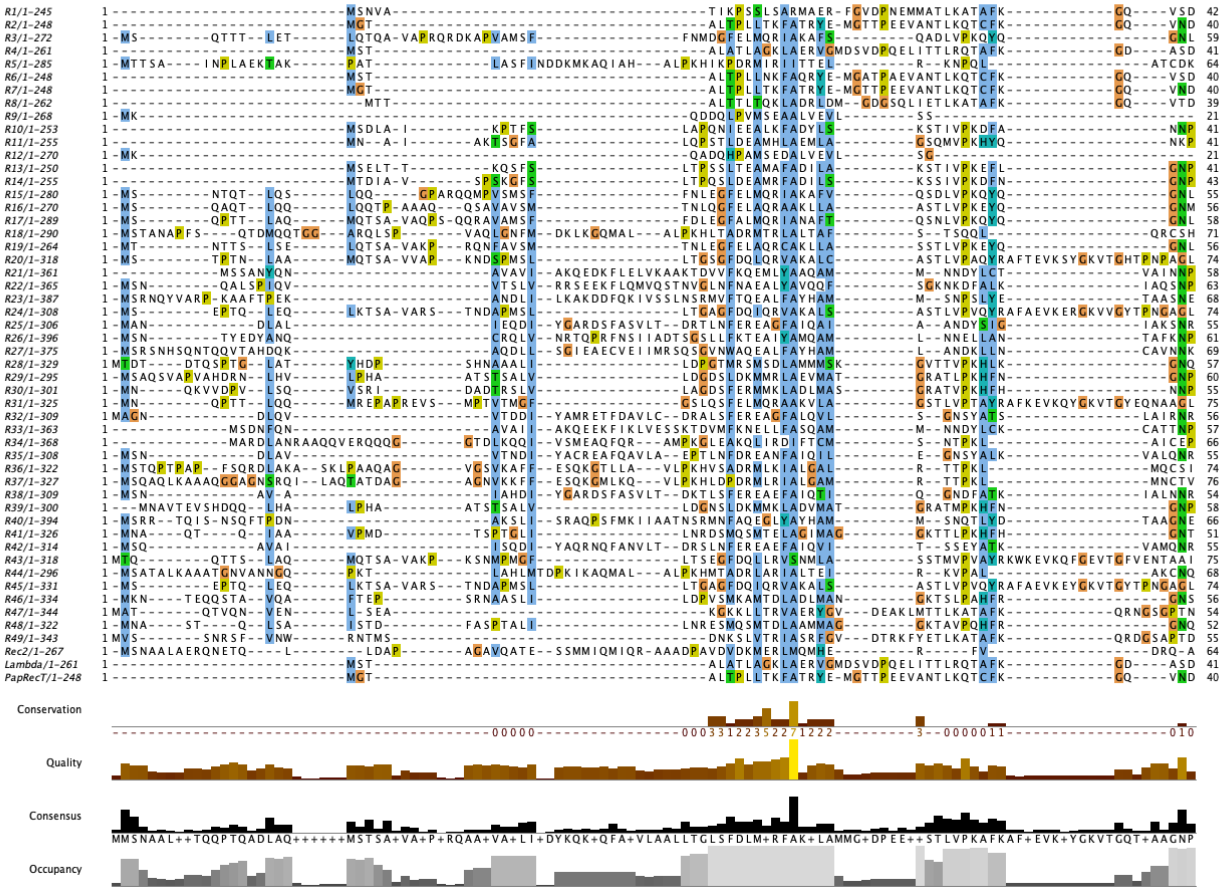

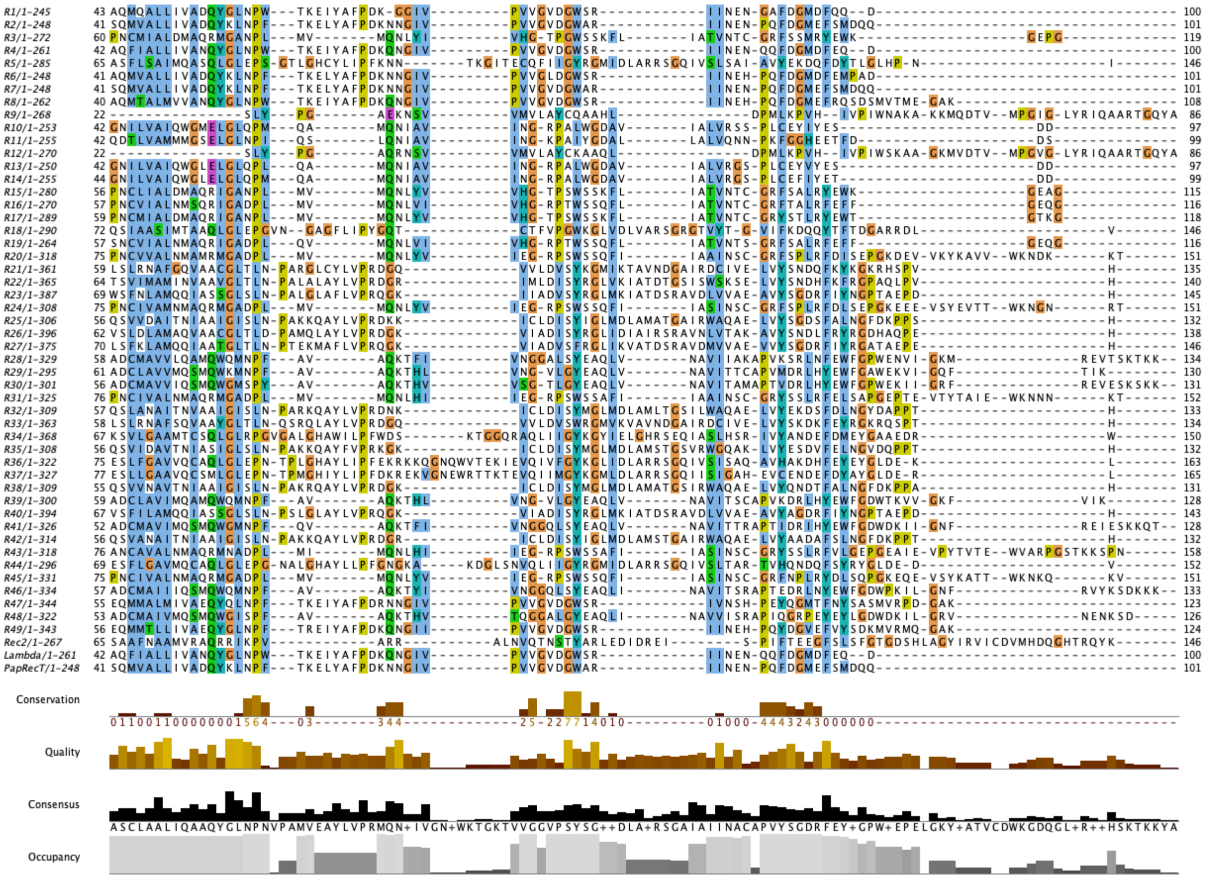
R1/1-245
R2/1-248
R3/1-272
R4/1-261
R5/1-285
R6/1-248
R7/1-248
R8/1-262
R9/1-268
R10/1-253
R11/1-255
R12/1-270
R13/1-250
R14/1-255
R15/1-280
R16/1-270
R17/1-289
R18/1-290
R19/1-264
R20/1-318
R21/1-361
R22/1-365
R23/1-387
R24/1-308
R25/1-306
R26/1-396
R27/1-375
R28/1-329
R29/1-295
R30/1-301
R31/1-325
R32/1-309
R33/1-363
R34/1-368
R35/1-308
R36/1-322
R37/1-327
R38/1-309
R39/1-300
R40/1-394
R41/1-326
R42/1-314
R43/1-318
R44/1-296
R45/1-331
R46/1-334
R47/1-344
R48/1-322
R49/1-343
Rec2/1-267
Lambda/1-261
PapRecT/1-248
Conservation
Quality
Consensus
Occupancy

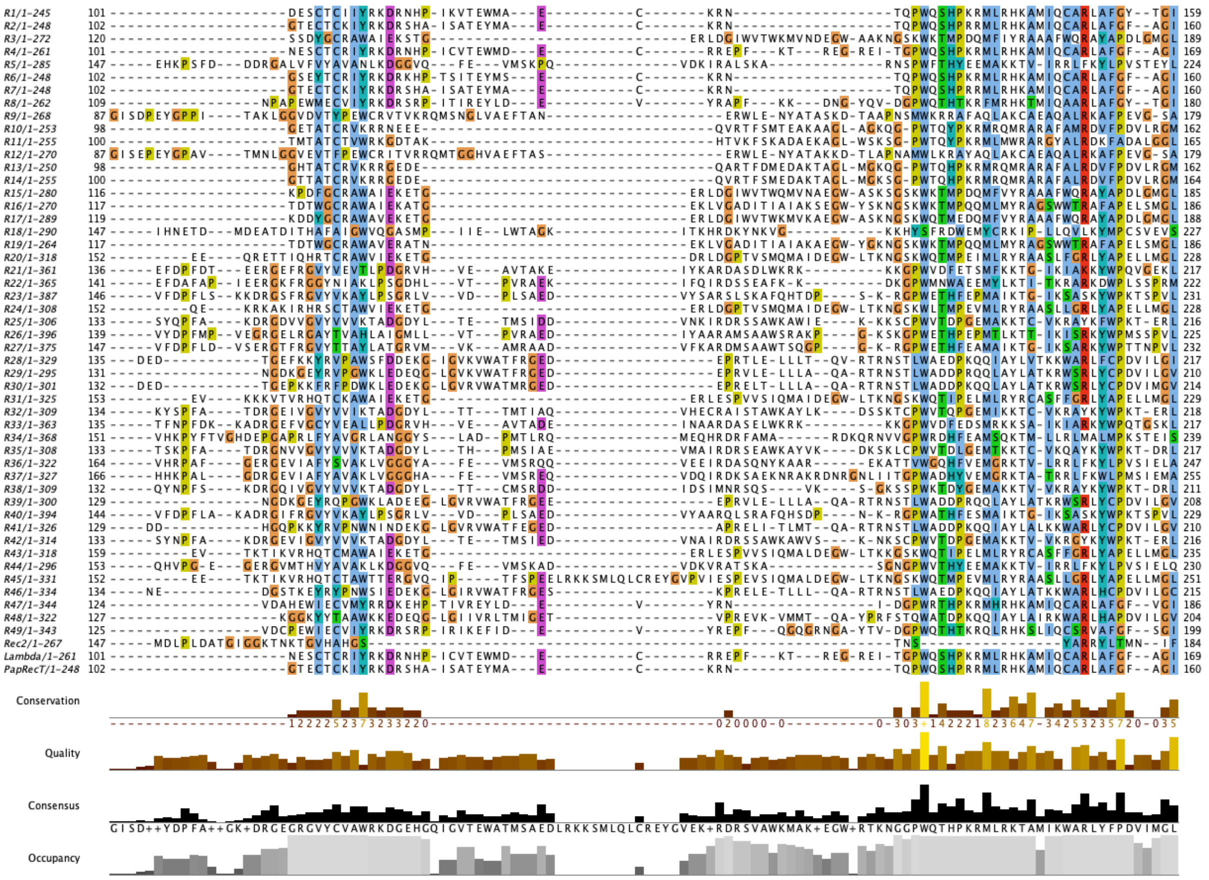

Conservation
Quality
Consensus
Occupancy

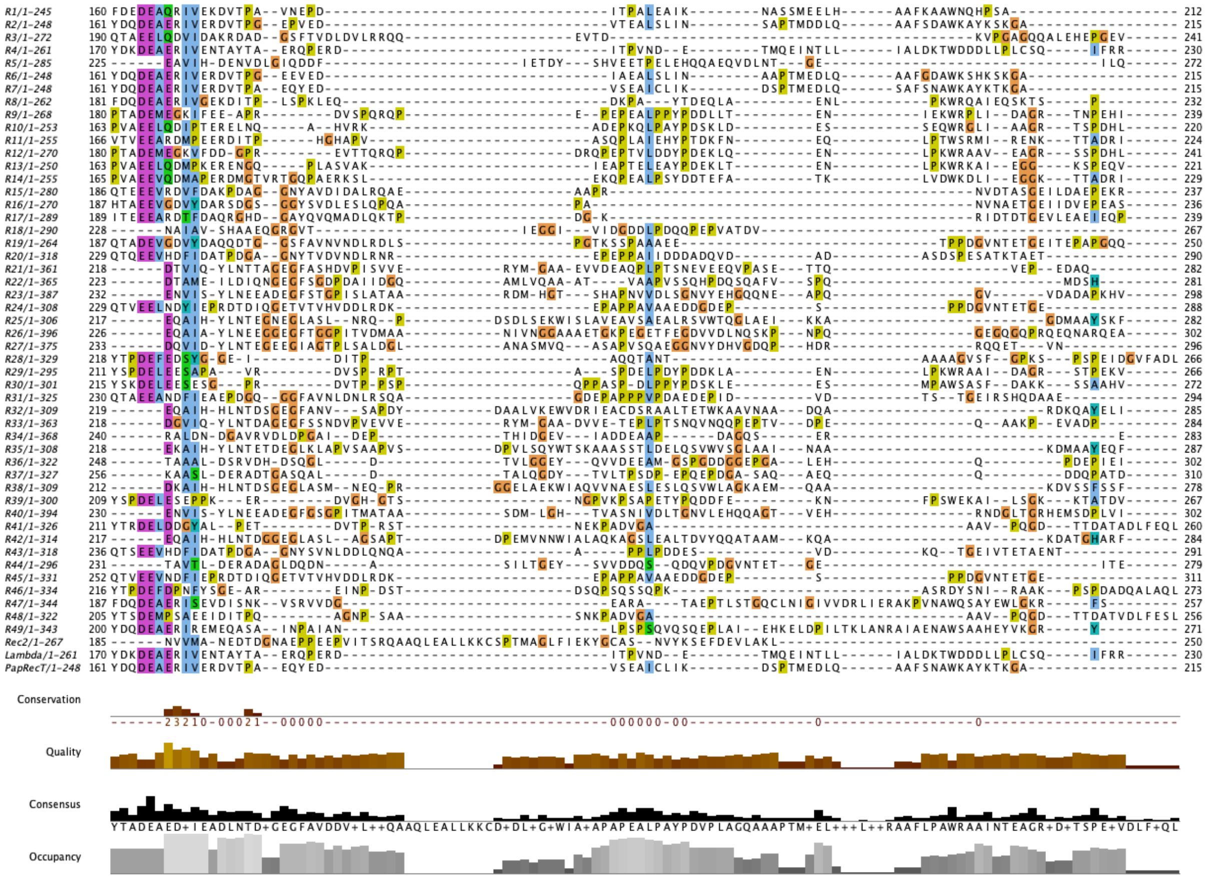
R1/1-245
R2/1-248
R3/1-272
R4/1-261
R5/1-285
R6/1-248
R7/1-248
R8/1-262
R9/1-268
R10/1-253
R11/1-255
R12/1-270
R13/1-250
R14/1-255
R15/1-280
R16/1-270
R17/1-289
R18/1-290
R19/1-264
R20/1-318
R21/1-361
R22/1-365
R23/1-387
R24/1-308
R25/1-306
R26/1-396
R27/1-375
R28/1-329
R29/1-295
R30/1-301
R31/1-325
R32/1-309
R33/1-363
R34/1-368
R35/1-308
R36/1-322
R37/1-327
R38/1-309
R39/1-300
R40/1-394
R41/1-326
R42/1-314
R43/1-318
R44/1-296
R45/1-331
R46/1-334
R47/1-344
R48/1-322
R49/1-343
Rec2/1-267
Lambda/1-261
PapRecT/1-248
Conservation
Quality
Consensus
Occupancy

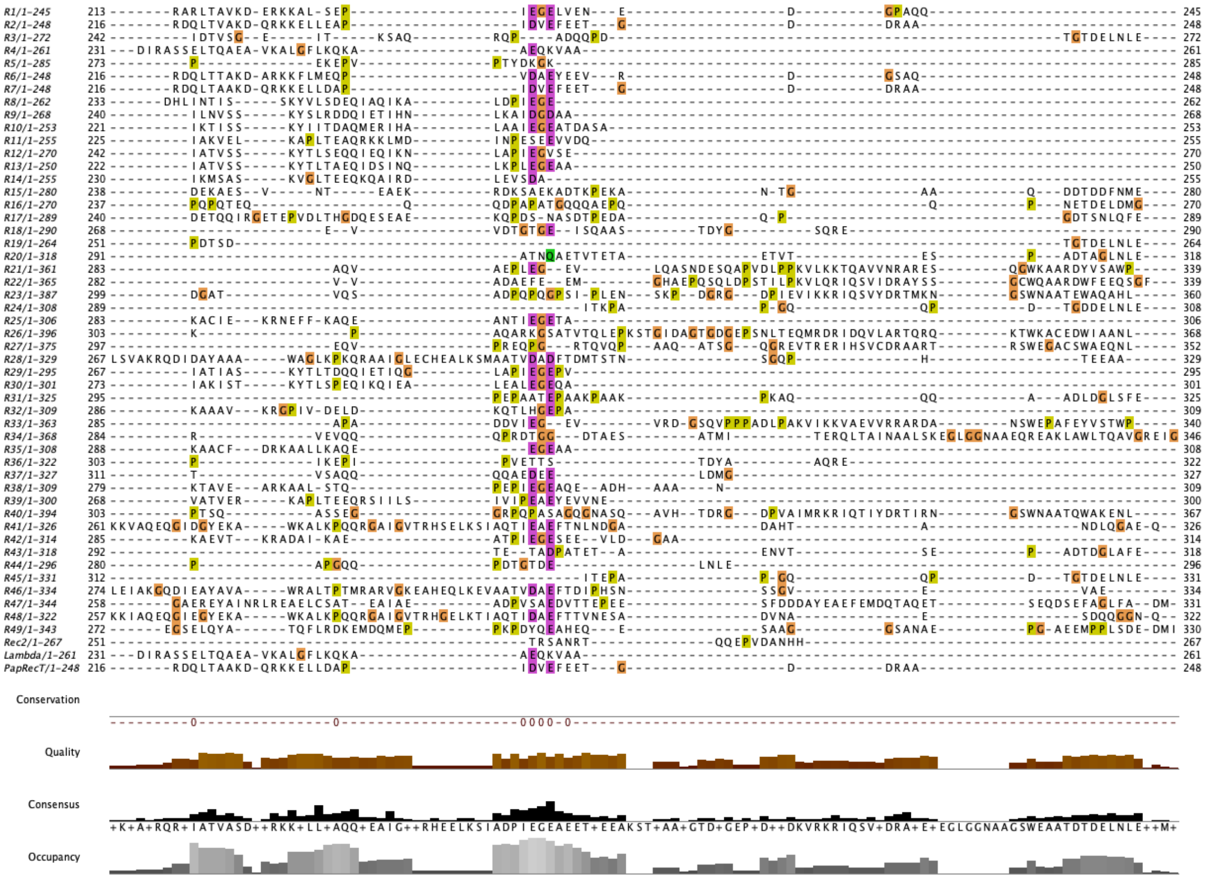
Conservation
Quality
Consensus
Occupancy

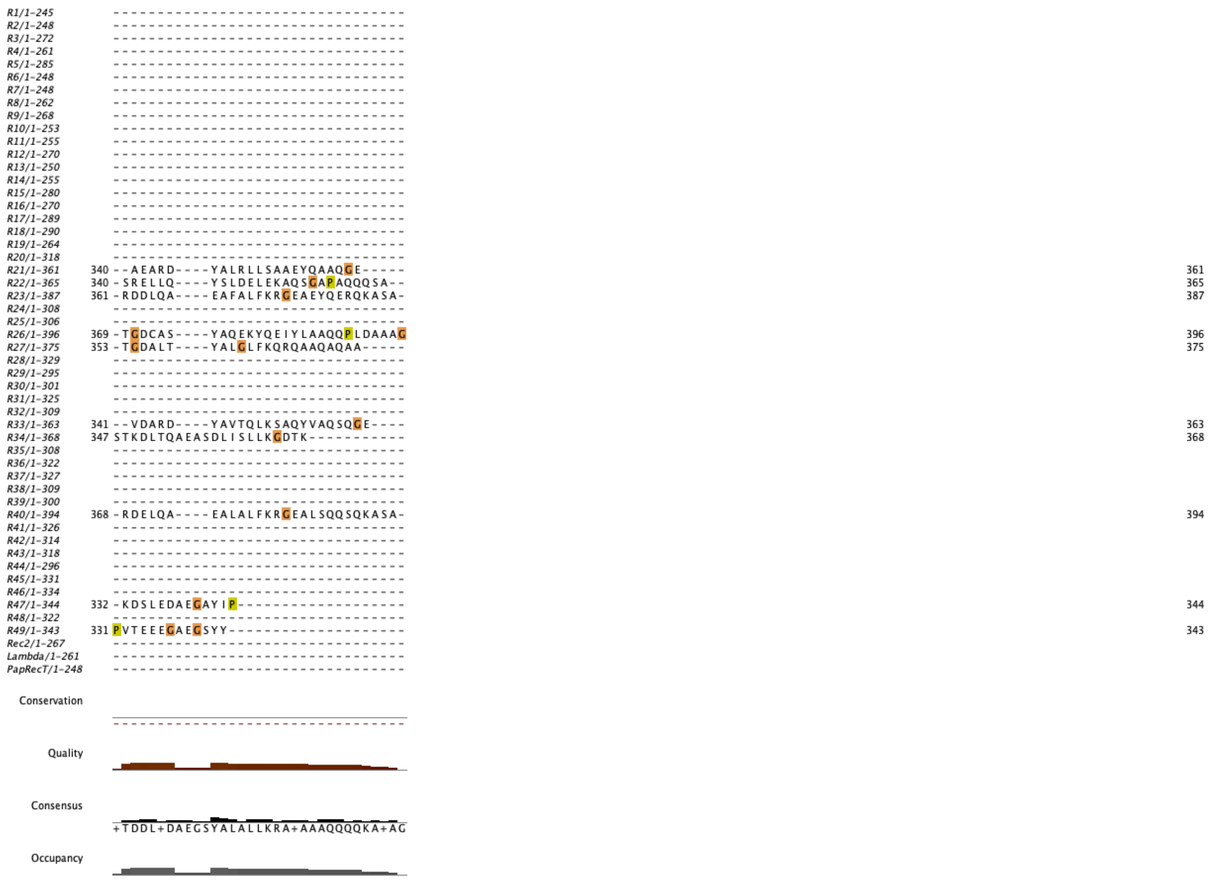

Supplementary Figure S1. Multiple sequence alignment of the 49 SSAP candidates and controls Rec2, PapRecT and Lambda Red β. MSA was done in Jalview 2.11.2.7 using ClustalO with default settings.

Supplementary Figure S2. Relative change of abundance of the SSAP candidates during the serial enrichment workflow in *P. putida* KT2440

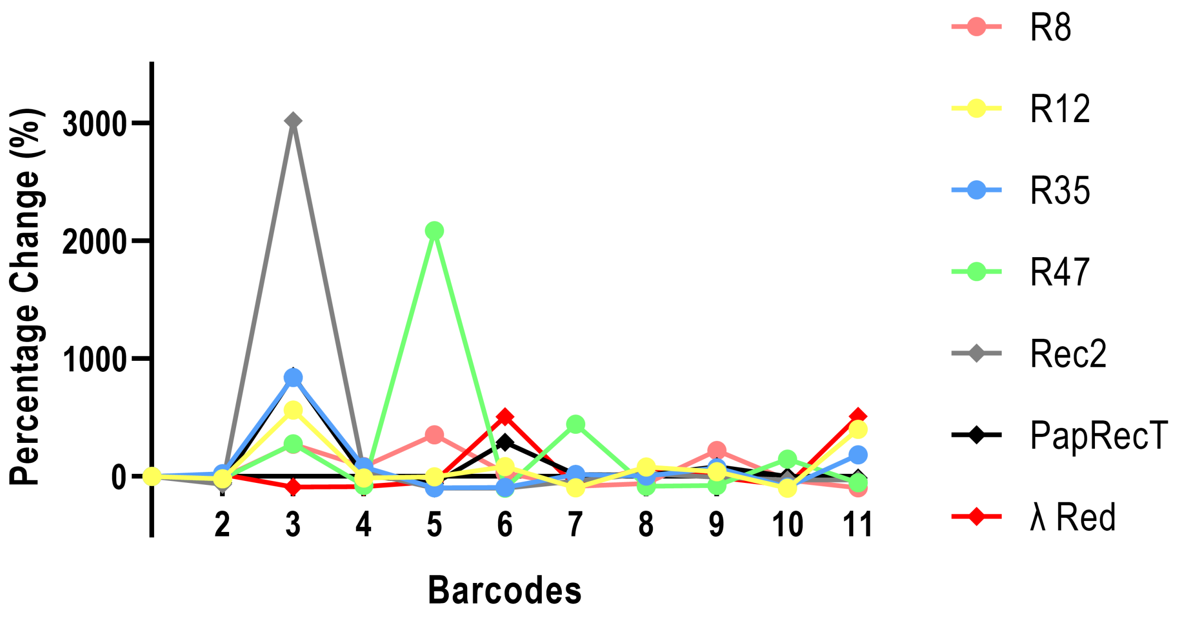

Supplementary Figure S2. Graph displaying the percentage change (increase and decrease) of the number of reads per recombinase compared to the previous cycle. To calculate percentage change, the difference (increase or decrease) between the percentages of a given recombinase within the whole population in two consecutive cycles was calculated. Then, the difference was divided by the percentage of that recombinases in the first of the two cycles (original number) and multiplied by 100.

Supplementary Figure S3. Profiles of the different growth curves of the individual *Pseudomonas* strains containing different SSAPs.

A

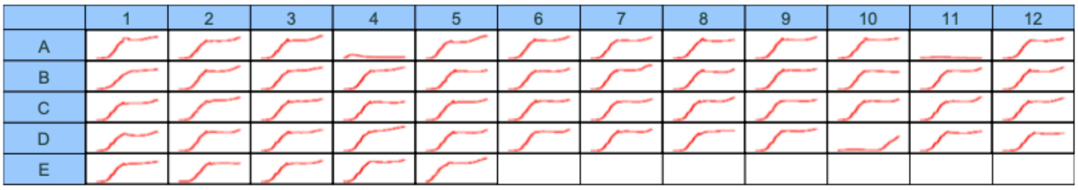

B

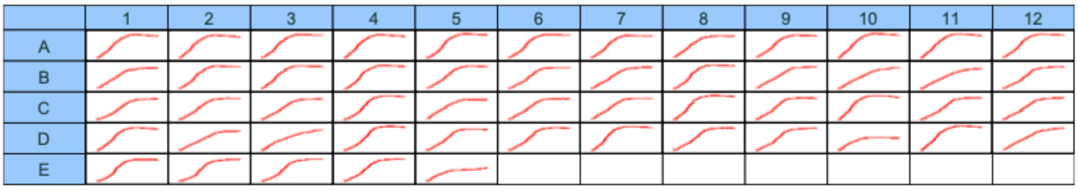

C

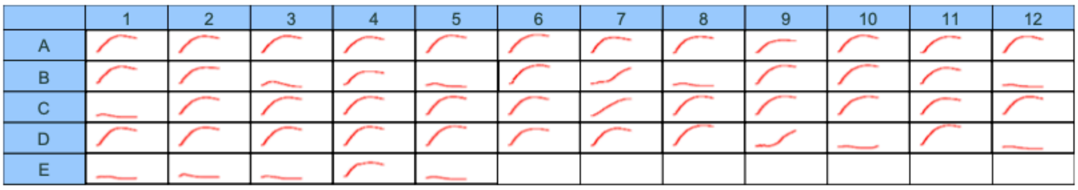

Supplementary Figure S3. Profiles of the growth curves of the individual *Pseudomonas* strains containing different SSAPs (A) *P. putida* KT2440, (B) *P. taiwanensis* VLB120, (C) *P. fluorescens* SBW25. Strains were grown at 30 °C and 200 rpm, overnight in 10 mL LB media supplemented with kanamycin. Cells were resuspended to an OD_{600} of 0.3 and grown aerobically at 30°C in fresh minimal LB medium supplemented with kanamycin at 50 µg/mL on 96-well transparent plates in a total volume of 200 µL per well. Optical density (OD_{600}) (y axis) was monitored in a BioTek Synergy Mx Multi-Mode Microplate reader over a 24 h period (x axis). Order of the samples starts with the strain containing the plasmid encoding R1 at A1 and continues from left to right and from top to bottom up to the strain carrying the plasmid encoding R49 at E1. The wells E2-E5 correspond to strains containing plasmids encoding Rec2, PapRecT, λ Red and an empty pSEVA2514-$mutL_{E36K}^{PP}$ vector, respectively.
